# Following Health Measures in the Pandemic: A Matter of Values?

**DOI:** 10.3389/fpsyg.2021.731799

**Published:** 2021-09-14

**Authors:** Carolin Schuster

**Affiliations:** Institute of Psychology, Leuphana University Lüneburg, Lüneburg, Germany

**Keywords:** values, moral behavior, behavioral intentions, COVID-19, social distancing

## Abstract

Three studies (*N* = 887) tested the hypothesis that value consistency predicts intended coronavirus disease-2019 (COVID-19) health behaviors and overrides other utility-based motivational factors. Accordingly, Study 1 showed that intentions of social distancing were higher if it was perceived as more value-consistent. The higher value consistency, the less self-interest inconsistency, and the perceived efficacy of social distancing mattered for intentions. On the other hand, Study 2 failed to induce value consistency experimentally. However, correlative results show a moderation pattern similar to Study 1 regarding social distancing intentions, policy support, and devaluation of transgressors. In Study 3, higher value consistency of vaccination reduced the experimental effect of prosocial efficacy but not the effect of self-interest efficacy of the vaccine. The findings are discussed regarding theoretical implications for the interplay of values and utility in motivation. In addition, implications for the potentially ambivalent effects of appealing to values to increase compliance are discussed.

## Introduction

The coronavirus disease-2019 (COVID-19) pandemic has demanded great sacrifices of people worldwide. Decision-makers and even individuals had to decide over the best course of action to avoid the spread of the virus throughout the year 2020 and ongoing. Preventive measures such as social distancing, wearing of masks, and lockdown policies have required citizens to restrict themselves in inconvenient and often personally costly ways. In addition, citizens have been demanded to comply despite the information about the effectiveness of health measures to flatten the curve of infections was yet unknown. However, individuals are motivated by the perceived efficacy of their behavior to achieve positive outcomes and minimize negative outcomes, e.g., Baumeister et al. (2007), Kahneman and Tversky (1979), and Vroom (1964). Given the uncertainties and hardships of the pandemic, fostering public compliance with health recommendations seems to be a major challenge. Compliance is demanded even from young, healthy individuals with lower risks to suffer gravely from an infection (Crimmins, 2020; Dowd et al., 2020). Since these individuals can still infect others with higher mortality or long-term health risks, compliance has often been discussed as a moral question of putting the public good above the self, as a matter of solidarity with others in society, and of protecting the lives of all people, e.g., Brakman (2020), Hinsliff (2020), and Kluger (2020).

Some politicians have urged citizens for compliance with health measures based on these values in their communications (Merkel, 2020; Queen Elizabeth, 2020). Pointing to the relevance and consistency of the behaviors of the individuals with their core values may be an effective instrument since values serve as guiding principles in the lives of the people (Schwartz, 1992; Sagiv et al., 2017). People strive to act in line with the core values they identify with (e.g., Bardi and Schwartz, 2003; Maio et al., 2009) and try to keep up their self-regard as moral persons (Zlatev et al., 2020). Nevertheless, this motivation might sometimes lead individuals to favor suboptimal options (Stöckli and Tanner, 2014; Zlatev et al., 2020). This research examines the role of perceived value consistency of health measures demanded from people in the pandemic for their intentions to comply. Specifically, the study shall examine whether value consistency is associated with the disregard of other motivating factors, such as personal costs or benefits associated with the health measures or the perceived efficacy of these measures. This study primarily contributes insights into the psychological processes underlying public compliance with social distancing demands, pandemic policies, and vaccination programs in the current COVID-19 pandemic. This knowledge is crucial for attempts to increase compliance with health measures. Furthermore, this research contributes to theory building on values as guiding principles by providing novel insights into the interplay of values and utility considerations for behavioral intentions. This knowledge may be applied to predict behavioral intentions in other societally relevant contexts, such as pro-environmental or prosocial behaviors.

## Values Guide Toward Consistent Behaviors

A multitude of studies shows that values are abstract goals that guide intentions, decisions, and behaviors across situations [for a review, see Sagiv et al. (2017)]. For instance, values predict according to voting behavior (Schwartz et al., 2010; Vecchione et al., 2013; Dennison et al., 2020), registering as an organ donor (Ryckman et al., 2005, 2009), and behavior in social dilemma games (Sagiv et al., 2011; Lönnqvist et al., 2013). Notably, self-transcendental and conformity values, which are typically understood as moral values (Schwartz, 2007), are important factors explaining the motivation of individuals for behavior that may be costly or effortful for the self but is for the sake of a greater good. This greater good may include pro-environmental behaviors (Stern et al., 1993; Karp, 1996; Thøgersen and Ölander, 2002) or altruistic allocation of resources in dilemma games (Sagiv et al., 2011; Lönnqvist et al., 2013). Similar values may also guide individuals to follow health measures in the COVID-19 pandemic, not only for their own sake but for the sake of others and overall society. It is important to note that a value can only guide the behavior of a person if they see how the abstract ideal of the value is connected to their own behavior. In other word, following a value means to behave in a certain way because this is what this value means [for a model of this link between value and behavior, see Ruepert et al. (2016)]. Based on the above reasoning, I hypothesise as follows.

H1: The extent to which individuals see health measures as consistent with their values predicts their intentions to comply.

There is evidence that consistent behaviors can be elicited by framing a situation in terms of values (Maio et al., 2009); this is often recommended as a strategy for campaigning for behavior change (Crompton and Kasser, 2009; Holmes et al., 2012; Brakman, 2020). In light of this, it seems completely reasonable to appeal to values such as solidarity and caring for others in the pandemic when asking people to reduce their physical contact with others.

## Values Are Non-Utilitarian Motivators of Behaviors

Framing a situation in terms of values may have ambivalent consequences. When making moral decisions, people may diverge from a rational approach of striving for optimal outcomes and ignore the potential benefits of other options (Berman and Kupor, 2020; Zlatev et al., 2020). During instances in conflicts that are framed as value-driven, negotiators seem to dismiss solutions that maximize joint outcomes for compromises with lower payoffs (Stöckli and Tanner, 2014) and reach lower outcomes for themselves and others (Schuster et al., 2020). Trying to act consistently with values in such conflicts increases personal involvement (Kouzakova et al., 2012) and motivates individuals to affirm their moral identity (Harinck and Ellemers, 2014), but not necessarily in a rational and effective way. When individuals perceive values as sacred, e.g., the protection of human life, they tend to ignore information about necessary trade-offs and avoid counterfactual thoughts (Tetlock et al., 2000; Tetlock, 2003). To keep up a self-view of living up to sacred values may require extreme personal sacrifices (Atran et al., 2014; Pretus et al., 2018). Sacred values are represented in the brain as non-utilitarian (Berns et al., 2012). The value relevance of following health measures in the current pandemic might be less extreme than that. However, other studies point toward a disregard for utilities even under conditions where the value motive was activated experimentally. Value-driven individuals avoided trade-offs in a negotiation even when this meant reaching less of their own valued goals (Schuster et al., 2020). In other studies, decision-makers mostly avoided trading off small risks or harms for outcomes with a higher potential moral payoff (Berman and Kupor, 2020; Zlatev et al., 2020). In the COVID-19 pandemic, value-driven individuals might be compliant and supportive to proposed measures no matter what the benefit or cost for the self. Additionally, they are obedient no matter what the efficacy of the compliant behavior in the context of the pandemic in general. Value-based compliance with health measures might thus override utilitarian considerations, such as costs, benefits, and expectancies associated with these measures. Based on the above grounds and discussion, this study hypothesizes as follows.

H2: Perceiving health measures as value consistent moderates the relationship of consistency with self-interests, i.e., cost or benefit of measures, with intentions to comply. The higher the value consistency of health measures, the lesser will be the influence of self-interest consistency.
H3: Perceiving health measures as value consistent moderates the relationship of perceived efficacy with intentions to comply. The higher the value consistency of health measures, the lesser will be the influence of efficacy.

## The Present Research

**Figure 1:**
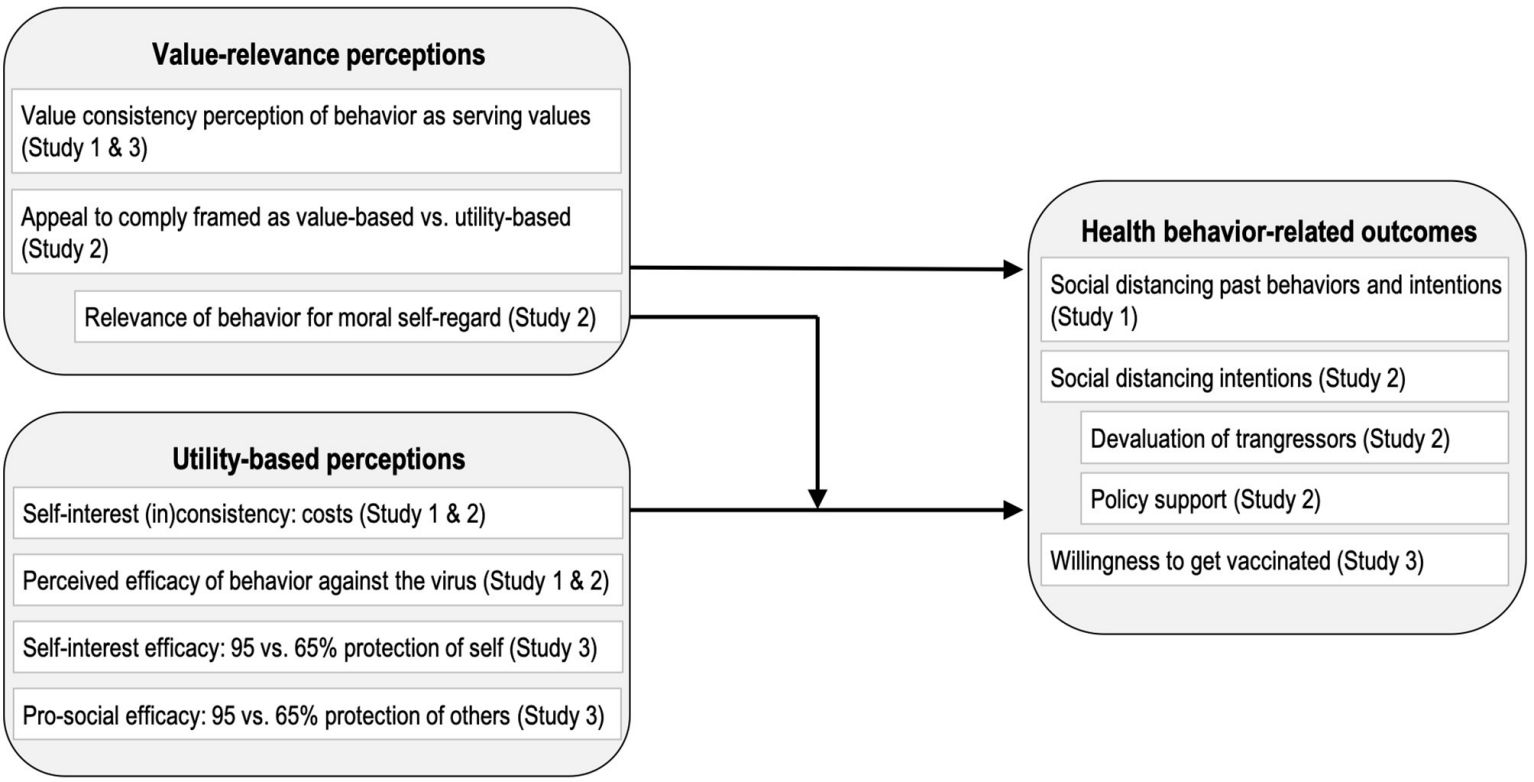
Overview of the research model and variables. Indented boxes display exploratory measures. Across studies, this study predicted that value-relevance perceptions moderate the extent to which utility-based perceptions explain health behavior-related outcomes.

The present study examines the role of values in three contexts: (1) the social distancing behavior of the individuals (Study 1, Study 2); (2) policy support of lockdown measures (Study 2); (3) vaccination intentions (Study 3) in the context of the COVID-19 pandemic. If the assumption that guidance by values at least partially overrides other motivational factors is correct, the perceived value consistency of behavior should predict the respective intention even beyond perceived personal benefits and costs (referred to as self-interests) and beyond the perceived efficacy of an individual in preventing the spread of the virus (referred to as efficacy). The more individuals feel guided by values on the issue, the fewer self-interests and efficacy should matter. Figure 1 provides an overview of the research model and the tested hypotheses.

Study 1 was conducted at the beginning of the first lockdown in Germany in March 2020. This study examined how value consistency affects social distancing behaviors and intentions and how it limits the respective roles of self-interest consistency and perceived efficacy. Study 2 was conducted in July across various English-speaking countries. The study aimed to test the hypotheses experimentally by testing the moderating effect of value-based compared to utility-based appeals to follow measures. Moreover, the study explores how moral self-regard considerations moderate the relevance of self-interest consistency and efficacy of social distancing measures for compliance as well as for the support of enforcing policies and for the devaluation of transgressors. Study 3 was conducted in October 2020 in a UK sample. This study focused on the willingness of individuals to get vaccinated with a hypothetical new vaccine. By experimentally manipulating self-interest efficacy, i.e., infection prevention, and prosocial efficacy, i.e., transmission prevention, causal conclusions can be drawn on how these utility considerations affect individuals while guided by values on the matter.

## Study 1: The Role of Value Consistency for Social Distancing

This research was conducted following the ethical guidelines of the American Psychological Association. Participation was voluntary and anonymous and could be terminated at any time. All measures are reported in the article. The original questionnaire and the data are available on the Open Science Framework (OSF; https://osf.io/fxv83/?view_only=5a76b0e3d5e948df84bc436aa6bd54fd).

### Method

#### Sample, Recruitment, and Design

The sample for this correlational study was recruited in Germany, between March 21 and April 24, 2020, *via* social networks, survey platforms, and university participant pools. They were invited to a survey about their attitude toward and experience with social distancing. Data collection started right before the federal government and the states issued guidelines about social distancing measures on March 22 (Bundesregierung Deutschland, 2020). A few days before, Chancellor Angela Merkel had urged every citizen in a televised speech to follow social distancing guidelines both for their own sake and for the sake of solidarity (Merkel, 2020). In total, 283 completed the study, 197 of which are women, 78 are men, and 8 are of other/undisclosed gender. Their age ranged from 16–72 with an *M* of 33.01 (*SD* = 13.82). Most participants were students (43.8%) and/or employed (40.3%).

#### Questionnaire and Measures

After giving informed consent, participants read a short introduction that stated the current call of politicians and health organizations to engage in social distancing to curb the spread of the virus. Then social distancing was explained as keeping distance from everyone outside the household of every person. To foster distancing, official decrees had closed schools, universities, and cultural institutions and shut down shops and other companies. Social distancing meant the demand to stay home as much as possible and reduce personal contacts to a minimum.

**Personal affectedness at work**. To better understand the sample, participants first reported how their workplace was affected by the situation. They chose between four options: At my workplace (1) everything is as usual, there is barely or no change, (2) I have to work more or in more challenging ways, (3) I do my tasks differently or restricted, e.g., home office, and (3) there is currently no or barely work, e.g., the shop is closed. Participants were further presented with a fifth option which is an open-ended option.

**Self-interest consistency**. Participants reported the extent to which social distancing was beneficial or harmful for themselves. The three items were introduced with “The more I socially distance, the more …” following 7-point semantic differentials (it has personal disadvantages for me—it has personal advantages for me; it is harmful to me—it is useful for me; it is in the way of my own interest—it serves my own interest). The combined scale was coded such that values > 0 indicate self-interest consistency and values <0 interest conflict (α = 0.872).

**Value consistency**. Participants indicated the extent to which compliance with social distancing was in line with or against their value convictions. The items were introduced with “If I socially distance as demanded, …” following 7-point semantic differentials (it completely contradicts my values—it completely corresponds with my values; I act against my conviction—I follow my conviction; I act fundamentally wrong—I act fundamentally right and good). The combined scale was coded such that values > 0 indicate value consistency and values <0 value conflict (α = 0.853). Following the questions on interest and value consistency, participants had the chance to leave comments.

**Related values**. On the next page, participants were asked to indicate which values they were referring to. They were asked to mark all their values that they felt the demand to socially distance was either serving or contradicting depending on whether they had indicated value consistency (>0) or inconsistency (<0) on the previous scale, respectively. Based on the value types from Schwartz' model (Schwartz, 1992), the following items including the explanation were listed: self-direction (i.e., to decide freely what to do), stimulation (i.e., to be stimulated by different outer influences), hedonism (i.e., to experience fun and pleasure), achievement (i.e., to reach something and make progress), power (i.e., to have influence), security (i.e., to protect the self and others), following to norms (i.e., to follow traditional or accepted rules and customs), the well-being of the people around oneself (i.e., to be considerate of them and support them), universal welfare (i.e., the equal respect for all humans and the planet as a whole), and an open-ended option for other values. The frequencies are reported in the Supplementary Figure 1.5.

**Perceived danger**. Exploratively, participants have indicated the danger they perceived from the virus for society on a scale ranging from 0 (no danger at all) to 100 (the highest possible danger). On this measure, most participants (>80%) only used steps of five to answer. Therefore, these scales were transformed by division through five and rounding to one integer (now ranging from 0 to 20).

**Perceived behavioral control**. Exploratively, participants answered on a scale from 0 to 100% how much they perceived their following of social distancing guidelines a matter of their own will (100%) or not at all if they felt they were being forced (0%); this scale was transformed to 1–20 for the same reason as explained above.

**Efficacy**. Participants indicated the extent to which they believed they could affect the outcomes of the crisis by following social distancing measures on a scale from 0 (no efficacy at all) to 100 (high efficacy), and this scale was also transformed to 1–20.

**Social distancing behavior**. The dependent variable was measured in a concrete and in a more general way. Participants were asked, concretely, how much they had engaged in several specific distancing behaviors during the past week, and how much they intended to do so in the following week. They answered on a 4-point scale ranging from 0 (I do not follow at all) to 4 (I follow completely). Besides, there was an option that the measures did not apply to oneself (e.g., because one's job did not allow it). The items were: 1. Hold at least 2 m distance to others; 2. Avoid touching, e.g., shaking hands; 3. avoid being in the same room with colleagues at work; 4. avoid contact with clients or customers at work; 5. avoid private events with more than 5 persons; 6. avoid private meetings, e.g., with friends; 7. avoid visiting public places like restaurants and gyms. The social distancing behavior scale was calculated as the sum of these items (excluding items 3 and 4, due to low applicability) for participants who answered them all for both weeks (i.e., 10 items, α = 0.865, values ranging from 0 to 30). The frequencies of each item and the reasoning for scale building are described in the Supplementary Table 1.1. Second, participants answered on a summary measure to what extent they, overall, comply with social distancing on a scale from 0 (not at all) to 100 % (follow completely), and this scale was transformed to 1–20.

**Demographic question**. Finally, participants indicated their gender, age, classification as a high-risk individual for COVID-19, e.g., elders, individuals with comorbidities, occupational status, highest educational degree, and the branch of their work. In the end, participants could again leave comments.

### Results

#### Preliminary Analysis: Social Distancing and Job Relevance

Participants were differently affected by the Coronavirus outbreak in their workplace. Aside from the participants who had not been working before, e.g., they were retired or on paternal leave, 9% of the participants said that there was no or barely any change at their workplace. There were 46% among the participants who worked on different tasks or their usual tasks in a restricted way, e.g., home office, digital classes, 13% could not /barely work, e.g., shop closed, and 7% had to work more than usual or in more difficult ways. A one-factorial ANOVA shows that this nominal factor did not significantly affect social distancing behavior, *F*_(4, 256)_ = 1.79, *p* = 0.132, nor the social distancing summary measure, *F*_(4, 273)_ = 1.43, *p* = 0.225.

#### Hypothesis Tests: Value Consistency as Moderator

The hypothesis that values may override self-interest and efficacy as predictors of social distancing was tested with bootstrapped moderation analyses with the SPSS PROCESS macro (Hayes, 2017; Model 1, 10,000 bootstrapped samples). Value consistency was entered as a moderator in all analyses and the social distancing measures as dependent variables. Table 1 shows the results of the moderation models for both social distancing measures. Value consistency explains unique variance in both distancing measures, and (mostly) moderates the effect of interests and efficacy on social distancing.

**Table 1:** The role of value consistency for social distancing.

	**Social distancing behaviors**			**Social distancing summary**		
	**Coefficient**	**95% CI [LL; UL]**	***p***	**Coefficient**	**95% CI [LL; UL]**	***p***
**H1 model**						
Self-interest consistency	0.542	[0.117; 0.966]	0.013	0.256	[−0.029; 0.541]	0.078
Value consistency	0.492	[0.160; 0.825]	0.004	0.434	[0.209; 0.659]	<0.001
Interaction	−0.253	[−0.432; −0.074]	0.006	−0.109	[−0.231; 0.013]	0.080
Age	0.004	[−0.036; 0.045]	0.827	0.026	[−0.002; 0.053]	0.071
Day	−0.060	[−0.098; −0.023]	0.002	−0.022	[−0.048; 0.003]	0.086
Risk group	1.481	[0.227; 2.736]	0.021	0.656	[−0.175; 1.487]	0.122
**H2 model**						
Efficacy	0.197	[0.084; 0.309]	0.001	0.243	[0.172; 0.314]	<0.001
Value consistency	2.083	[1.116; 3.050]	<0.001	1.778	[1.148; 2.406]	<0.001
Interaction	−0.095	[−0.154; −0.036]	0.002	−0.089	[−0.127; −0.051]	<0.001
Age	0.004	[−0.037; 0.044]	0.854	0.025	[−0.001; 0.052]	0.059
Day	−0.059	[−0.096; −0.021]	0.002	−0.017	[−0.041; 0.07]	0.171
Risk group	1.350	[0.096; 2.603]	0.035	0.613	[−0.165; 1.390]	0.122

*The table displays the results of bootstrapped models of the effects of (1) self-interest consistency and (2) efficacy on social distancing compliance, and their moderation by value consistency. Efficacy is coded ranging from 0 (no efficacy) to 20 (complete efficacy). Interest and value consistency of social distancing are coded such that 0 indicates neutral positions, −3 the highest inconsistency, and +3 is the highest consistency. Age, day of the survey, and being in a risk group (0 = no, 1 = yes) are included as covariates. Running the models without covariates reduces the overall explained variance of the models but does not change the conclusions substantially (see Supplementary Table 1.2)*.

**Figure 2:**
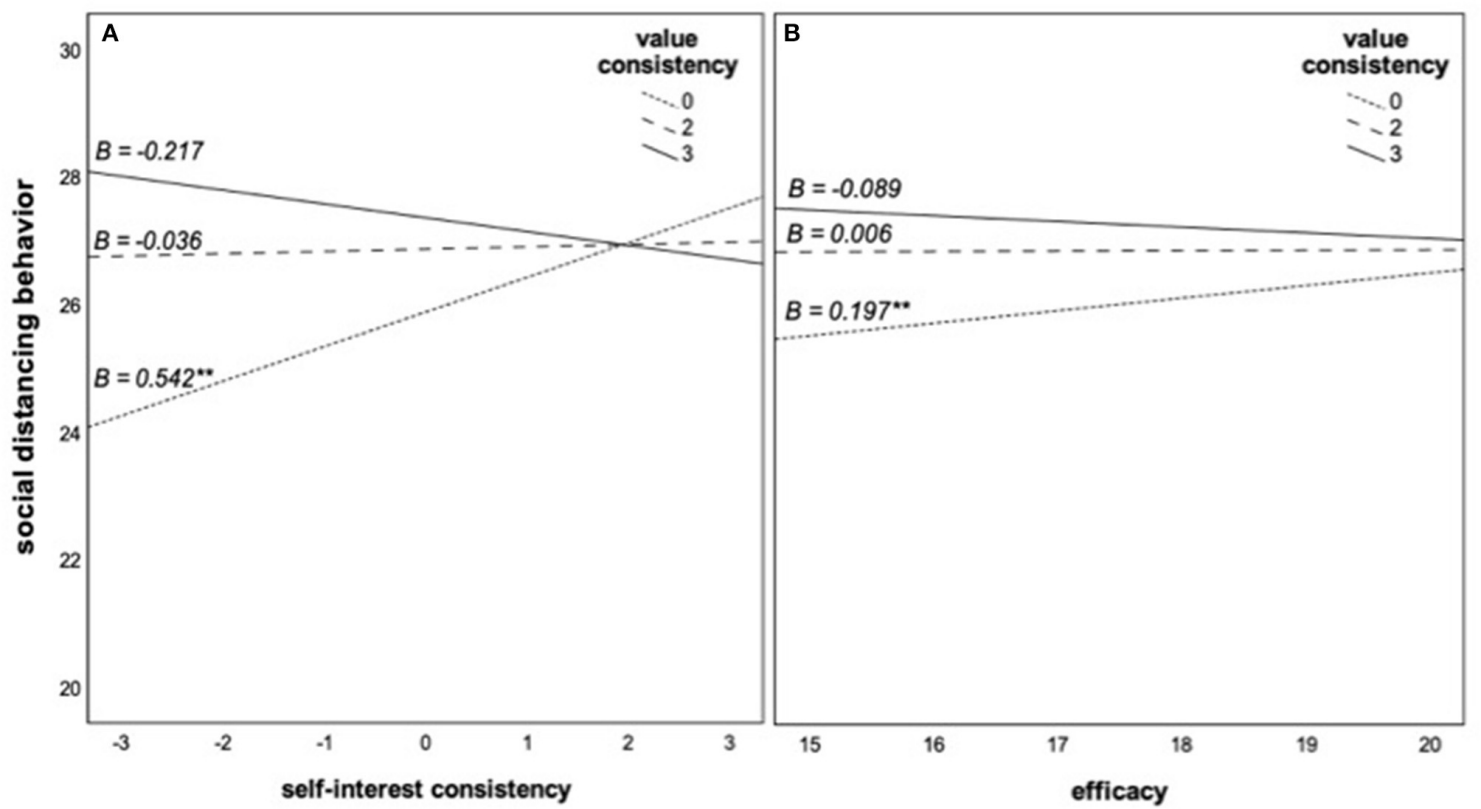
**(A,B)** Role of self-interest consistency and efficacy for social distancing behaviors at different levels of value consistency. The slopes represent the estimated conditional effects at the 16, 50, and 84th percentiles of the moderator value consistency. A value of 0 means that participants perceived social distancing as neither consistent nor inconsistent with their personal values. A value of 3 means that participants considered social distancing as completely consistent with their personal values. **p < 0.01.

Figure 2 shows that self-interest consistency (a) and efficacy (b) positively relate to social distancing behaviors among individuals with neutral value positions but not among those who feel social distancing is strongly in line with their values.

### Discussion

The study shows that the extent to which individuals see social distancing as consistent with their values is an important factor to explain compliance. A strong value consistency relates to the lower relevance of other predictors of behavioral intentions, such as personal benefits or costs or the perceived efficacy of the behavior. Given the convenience sample and the correlative design, the study can only be a first step in understanding the relationship between these variables and not inform about the causality of the effects.

## Study 2: Value Framing and Moral Self-Regard as Moderators

Study 2 was constructed to expand the findings from Study 1 in three major ways. First, the aim was to examine whether value-based appeals compared to utility-based appeals can causally elicit the kind of moderating effects of the role of self-interest and efficacy for compliance with social distancing. Second, a measure of moral self-regard in case of non-compliance was included as a potential mediator to further support assumptions about the psychological meaning of values. In different domains, it has been argued that values and moral principles lead to according behaviors because individuals identify with their respective values and morality and they feel threatened in their self-view if they act in a way that could be interpreted as a transgression, e.g., Harinck and Ellemers (2014), Hitlin (2003), Tetlock et al. (2000), van der Werff et al. (2013), and Zlatev et al. (2020). Therefore, value-based appeals might activate moral self-regard and thereby moderate the role of self-interest and efficacy on behavioral intentions. Third, the study aimed to examine if similar processes affect another outcome, the support of policies that enforce distancing measures. Previous research has shown that core values reflect in political attitudes and voting behavior (Schwartz et al., 2010; Vecchione et al., 2013). It is thus very likely that value consistency affects policy support. However, it is unclear whether value consistency also moderates self-interest and efficacy effects.

Given the experimentally manipulated appeal to comply with health-relevant behaviors, this study applied for voluntary ethics review, which was approved by the Ethics Review Board of the [#BLINDED] University. The experimental hypotheses that value-based appeals affect intentions (H1) and moderate the effects of self-interest consistency (H2) and efficacy (H3) on intentions to comply were pre-registered and are available on the project page in the OSF. All measures are reported in the article.

### Method

#### Sample, Recruitment, and Design

In total, 346 participants were recruited in July 2020 on Prolific.ac to a survey about their attitude toward and experience with social distancing. Six participants failed a treatment check and were excluded. The final sample consisted of 118 women, 218 men, and four of other/undisclosed gender (*N* = 340). The majority was currently living on the European Continent (67%) or in the United Kingdom (11%). Regarding their educational level, most participants have completed their general education on an advanced level (39.1%) or received an undergraduate degree (32.9%). The design is experimental with the factor type of appeal (value-based vs. utility-based).

#### Procedure

After giving informed consent, participants read a short introduction that stated that since the beginning of the COVID-19 pandemic, scientists and politicians had called the public to engage in social distancing and hygiene measures to minimize the spread of the coronavirus. The study was about these measures, which were subsequently listed. The nine measures were “wearing a face mask where recommended,” “avoiding crowded outdoor places,” “avoiding crowded indoor places,” “self-isolation in case of typical symptoms,” “avoiding physical contact,” “avoiding traveling by plane or public transport,” “using voluntary apps for contact tracing,” and “regularly disinfecting or thoroughly washing hands,” Subsequently, the interest inconsistency, i.e., personal costs, perceived efficacy, and perceived behavioral control over compliance were measured for each of the nine corona measures. An attention check was included here. Afterward, participants were randomly assigned to either a value-based or a utility-based type of appeal, which was allegedly formulated by a group of epidemiologists, doctors, and politicians in order to call attention to the need to curb the spread of the COVID-19. The appeal read:

“*Dear fellow citizens*,

*The ongoing pandemic poses a major challenge to all of us and many of you may be fed up with the demands for social distancing and extensive hygiene measures. However, there is still a great danger from this virus. The measures required of you are to prevent [value condition: endangering lives by] a further spread of the virus. They are based on [value condition: the core values of caring for everyone's well-being and protecting the people around you/utility condition: the latest research to prevent the risk of infection]. It is, therefore, a matter of [value condition: solidarity and compassion with our most vulnerable fellow human beings/utility condition: common sense and necessary for the functioning of the healthcare system] to comply with these measures until an effective vaccine has been developed or we are able to treat Covid-19 much more effectively. Please follow these [value condition: worthy/utility condition: reasonable] measures strictly and contribute to the containment of the virus.”*

Participants then reported their intentions to comply with each measure and were asked whether they would like more information about scientific evidence about the measures. Finally, a questionnaire followed including measures of pandemic policy support, personal values, regulatory focus, moral self-regard, devaluation of non-compliance of others, social norms of compliance, and demographic questions.

#### Measures

**Perceived self-interest inconsistency**. Participants were first asked how much each of the nine measures fitted or contradicted their self-interests (α = 0.782). Participants indicated for each measure the extent to which it was very costly (−3) to not costly at all (3).

**Perceived efficacy**. Participants were asked to indicate to what extent they believed they could personally contribute to curbing the spread of the virus by following each of the nine health measures (α = 0.883) on a scale of 0 (no impact at all) to 6 (very high impact).

**Perceived behavioral control**. Participants answered on a scale of 0 (no control at all) to 6 (full control) regarding how much they perceived their following of each of the nine measures (α = 0.752) as a matter of their personal choice.

**Behavioral intentions**. The dependent variable was measured by asking the participants how much they planned to follow each of the nine measures over the next month (α = 0.782). They answered on a 7-point scale ranging from 1 (not at all, never) to 8 (completely, all the time). Similar to Study 1, participants also answered in Study 2 a summary measure to what extent they intended to follow the measures overall on a scale ranging from 0 (not at all) to 100% (follow completely). As an exploratory behavioral indicator, participants were also asked if they would like to get more information about the scientific evidence on different measures to curb the further spread of the Coronavirus.

**Policy support**. Participants were asked on the next page to indicate how much they supported several specific policies in case of a renewed rapid increase of infections. They answered on a 6-point scale ranging from 1 (strongly oppose) to 6 (strongly support). The items were: 1. Enforcing the wearing of face masks indoor in public; 2. Restricting indoor group gatherings, e.g., private parties; 3. Restricting outdoor group gatherings, e.g., outdoor concerts, stadium attendance; 4. Prescribe self-isolation as mandatory/obligatory in case of typical symptoms; 5. Restricting unnecessary private trips; 6. Restricting the use of public transport and planes; 7. Making the use of contact tracing applications mandatory; 8. Temporarily closing schools; 9. Shutting down businesses (α = 0.863).

**Personal values**. Participants were then asked to indicate how important specific values are to them as a live-guiding principle on an 8-point scale ranging from 1 (not important) to 8 (of supreme importance). Furthermore, there was an option for participants to indicate if one item is opposed to their principles (coded as missing). The values are the 10 items from the Short Schwartz' Value Survey (SSVS; Lindeman and Verkasalo, 2005) and contain a value type and its description, e.g., benevolence (helpfulness, honesty, forgiveness, loyalty, and responsibility). The value items are analyzed separately.

**Regulatory focus**. This exploratory measure was included to examine whether the value framing induced a prevention focus. It consisted of three items (α = 0.632), which are described in the Supplementary Material 2.1.

**Moral self-regard**. Additionally, participants were asked to indicate how they would feel in case of not complying with the health measures. The three items (α = 0.909) were as follows: (1) To what extent would you feel like a bad person?; (2) To what extent would you feel less moral than you would like to feel?; (3) To what extent would you feel you were acting against your own values? (Zlatev et al., 2020). Participants answered on a 6-point scale ranging from 0 (not at all) to 5 (very much). This measure thus represents the linking of the value-based identity of a person with their behavior regarding the health measures. Therefore, it is vital for the process by which moral values regulate behavior.

**Devaluation of transgressors**. This exploratory measure assessed the reactions of the participants to observing other people not following the health measures in the next month. It consisted of the following six items (α = 0.856): (1) To what extent would you perceive them as a bad person?; (2) “To what extent would you perceive this as immoral behavior?; (3) To what extent would you perceive them as acting against your values?; (4) To what extent would you try to let them feel your disapproval?; (5) To what extent would you have an impulse to scold them?; (6) To what extent would you like to avoid them?” The scale ranged from 1 (not at all) to 6 (very much).

**Social norm**. Participants rated three items on how they expected people around them to judge their behavior regarding the health measures on 6-point scales. The items are reported in the Supplementary Material 2.1. Due to technical problems, answers on these items were not recorded correctly and they were not analyzed.

**Demographic questions**. Finally, participants indicated their age, gender, highest educational degree, current place of residence, classification as a high-risk individual of COVID-19, e.g., elders, individuals with comorbidities, if they have personal experience with a COVID-19 infection, e.g., self, among personal contacts, and to what extent their job situation has been negatively affected by lockdown and preventive measures.

### Results of Study 2

#### Preliminary Analyses

Following the pre-registered procedure, correlations of the demographic variables with behavioral intentions were computed (for complete intercorrelations, see Supplementary Table 2.2). Age, experience with infection, and (binary-coded) gender correlated significantly with either the generalized measure or the mean specific behaviors measure or both, *r*_age_ = 0.165/0.134, *p* = 0.002/0.013, *r*_inf_ = 0.117/0.098, *p* = 0.031/0.071, *r*_gender_ = 0.150/0.093, *p* = 0.006/0.087, respectively. These variables were consequently included as covariates in the analyses of behavioral intentions.^1^ In addition, the two behavioral intention indicators (the generalized compliance item and the mean behavioral intentions for specific measures) correlated highly, *r* = 0.65, *p* < 0.001. Since the results of the following analyses do not support different conclusions when using the generalized item, this study will focus on the results regarding the specific behavioral intentions.

#### Confirmatory Hypothesis Tests: Type of Appeal Makes No Difference

A full report of the pre-registered experimental hypothesis tests can be found in the Supplementary Material 2.4. In the following, they were only summarized and the study focused on the more interesting explorative analyses. A *t*-test showed that the framing condition had a significant effect on the manipulation check item about value-based motivation, *t*_(337.71)_ = −2.8, *p* = 0.005, such that participants perceived the authors to be more motivated by values in the value framing condition, *M* = 4.91, *SD* = 1.49, compared to the neutral framing condition, *M* = 4.43, *SD* = 1.63. The perception of rational thought as motivation was high in the value framing condition, *M* = 5.94, *SD* = 1.31, as well as in the neutral framing condition, *M* = 5.7, *SD* = 1.29, with no significant difference, *t*_(338)_ = 1.70, *p* = 0.09.

Even though the framing manipulation seemed to have been noticed by participants, the appeal did not affect their behavioral intentions. ANOVAs controlling for the covariates, showed no significant framing effect on specific behavioral intentions, *F*_(1, 331)_ = 0.11, *p* = 0.745. In addition, the framing condition did not moderate the effect of perceived self-interest inconsistency on behavioral intentions, *B* = 0.601, *p* = 0.184, Δ*R*^2^ = 0.004, nor the effect of perceived mean measures efficacy on behavioral intentions, *B* = −0.053, *p* = 0.550, Δ*R*^2^ < 0.001. Multi-level analyses that tested whether the framing condition (on the personal level) affected the extent to which self-interest inconsistency and efficacy on the measure level explain intentions to comply similarly do not support any main or moderating effect of framing (see Supplementary Table 2.4). Therefore, all of the pre-registered hypothesis tests about the framing condition's effects have to be rejected.^2^

#### Exploratory Analyses: Moral Self-Regard as an Indicator for Value Consistency

**Figure 3:**
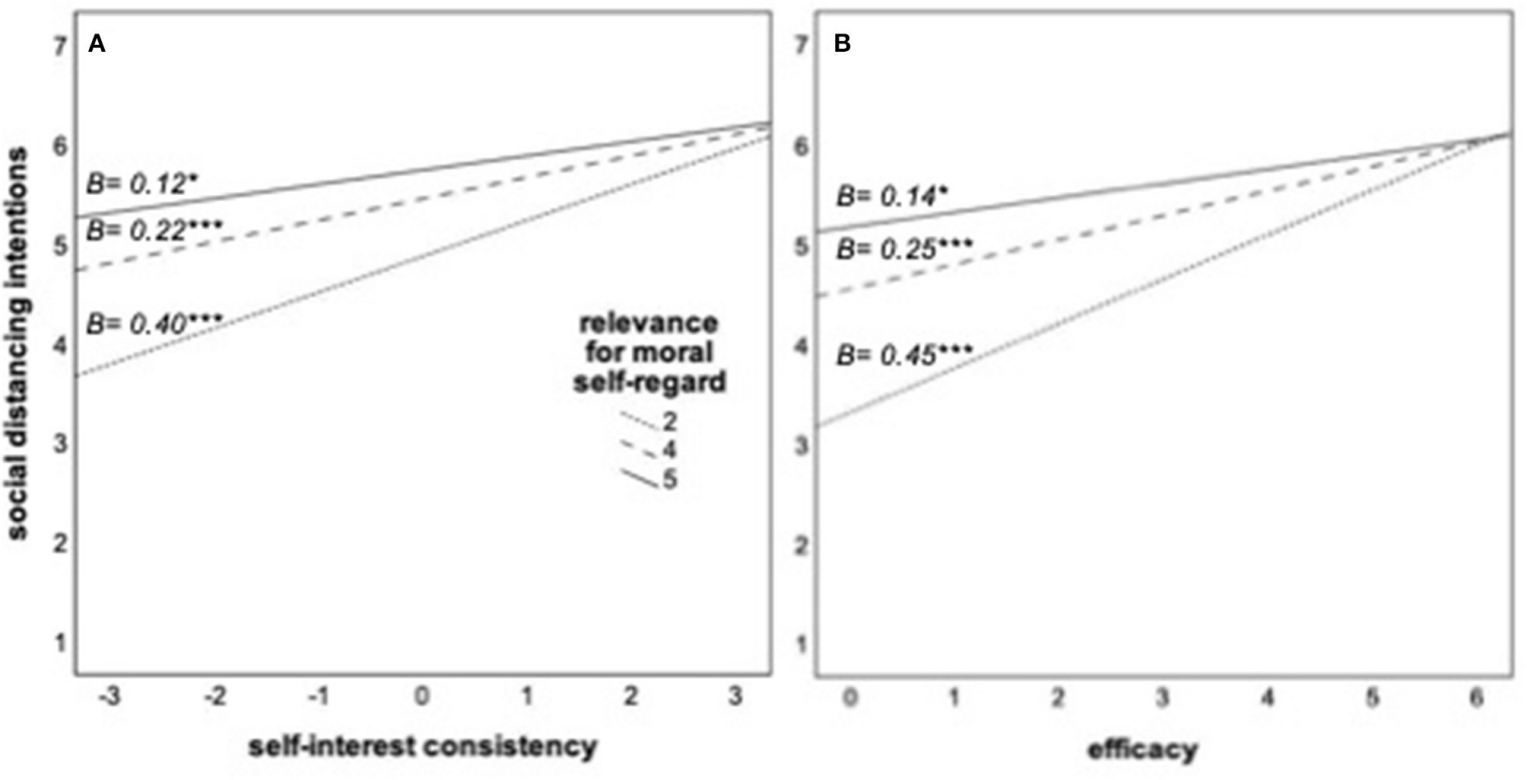
**(A,B)** Role of self-interest consistency and efficacy of health measures for social distancing intentions at different levels of the relevance of complying for moral self-regard. The slopes represent the estimated conditional effects at the 16, 50, and 84th percentiles of the moderator. *p < 0.05; ***p < 0.001.

The hypotheses were based on the theoretical assumption that interpreting behavior as relevant for and consistent with core values makes the behavior relevant for the identities of the individuals, thus motivating them to act accordingly. The appeal was effective in communicating the value relevance of social distancing for the authors of the appeal, as the manipulation check shows. However, the value framing of appeal did not lead to a significantly stronger implication of self-regard after transgressions, *M* = 3.59, *SD* = 1.2, than the neutral framing, *M* = 3.39, *SD* = 1.43, *F*_(1, 338)_ = 1.32, *p* = 0.189. To explore the theoretical assumptions behind the original predictions further, this study analyzed the data substituting the experimental value framing with the measure of self-regard (Table 2). In line with the theoretical reasoning, the relevance of the measures for the self-regard of a person significantly predicted behavioral intentions beyond the covariates and the effect of self-interest consistency and moderated the effect of self-interest consistency. Similarly, the self-regard measure moderated the effect of efficacy on behavioral intentions beyond the significant main effects of self-regard and efficacy. Figure 3 shows the conditional effects.

**Table 2:** Interaction effects of moral self-regard and self-interest consistency (Model 1) and efficacy (Model 2) on measures of social distancing intentions.

	**Social distancing intentions**			**Social distancing summary**		
	**Coefficient**	**95% CI [LL; UL]**	***p***	**Coefficient**	**95% CI [LL; UL]**	***p***
**Model 1**						
Self-interest consistency	0.591	[0.373; 0.808]	<0.001	8.683	[4.695; 12.672]	<0.001
Self-regard	0.288	[0.208; 0.368]	<0.001	4.880	[3.414; 6.345]	<0.001
Interaction	−0.094	[−0.151; −0.036]	0.001	−1.643	[−2.699; −0.588]	0.002
Age	0.020	[0.010; 0.030]	<0.001	0.298	[0.114; 0.481]	0.002
Gender	0.165	[−0.017; 0.347]	0.076	1.060	[−2.276; 4.397]	0.532
Infection	0.130	[−0.064; 0.324]	0.188	1.729	[−1.827; 5.286]	0.339
**Model 2**						
Efficacy	0.647	[0.471; 0.823]	<0.001	8.707	[5.395; 12.020]	<0.001
Self-regard	0.319	[0.226; 0.412]	<0.001	10.265	[6.016; 14.513]	<0.001
Interaction	−0.101	[−0.150; −0.051]	<0.001	−1.612	[−2.545; −0.680]	0.001
Age	0.017	[0.008; 0.027]	<0.001	0.262	[0.081; 0.443]	0.005
Gender	0.087	[−0.088; 0.261]	0.331	0.163	[−3.123; 3.448]	0.923
Infection	0.119	[−0.068; 0.306]	0.210	1.685	[−1.829; 5.198]	0.346

*Efficacy is coded ranging from 0 (no efficacy) to 6 (complete efficacy). Self-interest consistency of social distancing ranges from−3 (very inconsistent/costly) to 3 (very consistent/beneficial). Self-regard ranges from 0 (no relevance for self-regard) to 6 (high relevance for self-regard). Running the model without the covariates age (standardized), previous infection in social circle (0, no; 1, yes), and binary gender (0, male; 1, female) does not change the conclusions (see SOM)*.

**Figure 4:**
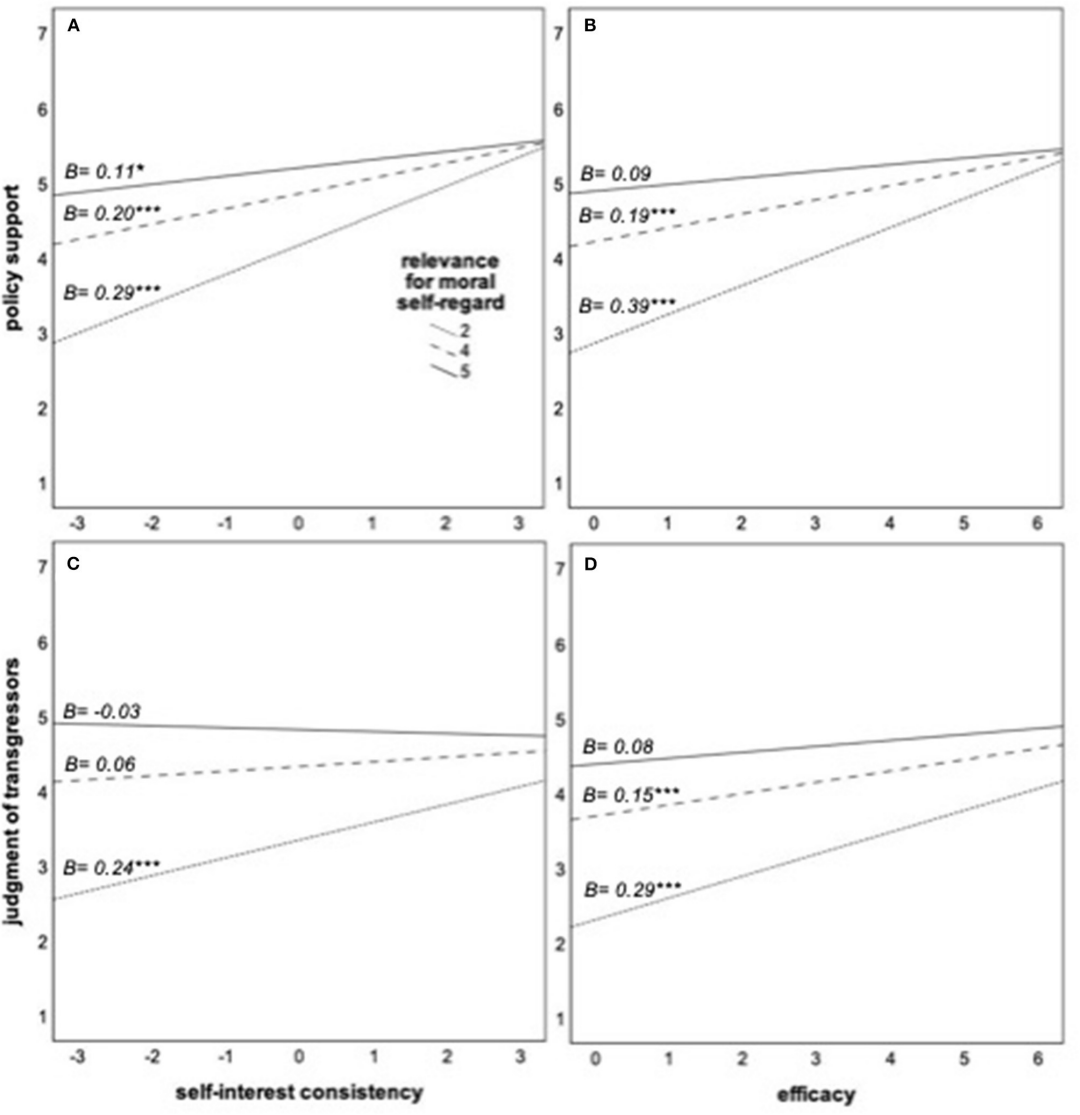
**(A–D)** Role of self-interest consistency and efficacy of health measures for enforcement policy support and judgment of non-compliant others at different levels of the relevance of complying for moral self-regard. The slopes represent the estimated conditional effects at the 16, 50, and 84th percentiles of the moderator. *p < 0.05; **p < 0.01; ***p < 0.001.

In addition, four additional moderation models^3^ with the same predictors (self-regard and either self-interest consistency or efficacy) and policy support and judgmental reactions to the transgressions of others as dependent variables were calculated. Self-regard significantly moderates the effects of self-interest consistency, *B* = −0.094, *p* = 0.001, Δ*R*^2^ = 0.023, as well as of efficacy, *B* = −0.099, *p* < 0.001, Δ*R*^2^ = 0.032, on policy support; and of self-interest consistency, *B* = −0.088, *p* = 0.006, Δ*R*^2^ = 0.015, and of efficacy, *B* = −0.071, *p* = 0.011, Δ*R*^2^ = 0.012, on judgmental reactions. As Figures 4A–D shows, the pattern is always the same: how costly or effective social distancing measures are perceived explains policy support to enforce them and judgmental reactions to the transgressions of others less among participants whose own moral self-regard is linked to their social distancing.

Explorative analyses of the correlations of the outcomes with specific values show that the strongest link seems to be with benevolence values, such that behavioral intentions (specific and general), policy support, devaluation of transgressors, and moral self-regard all correlate positively with this value type, *r*s > 0.163, *p*s <0.003. Regarding the other values, the correlations are less consistent with a tendency for positive correlations with security and universalism and negative correlations with power and hedonism (complete correlations in Supplementary Table 2.1).

### Discussion

Study 2 shows that contrary to the pre-registered hypotheses, framing an appeal to follow COVID-19 health measures as based on benevolence values, compared with framing it as based on reason, neither directly affected behavioral intentions nor moderated the role of the self-interest inconsistency of the measures for the self and efficacy in the pandemic. In previous studies, value framing was effective to change the behavior of the participant, e.g., Kouzakova et al. (2012), Maio et al. (2009), and Schuster et al. (2020). Even though framing may sometimes affect how individuals see connections between their values and specific behavior, the present study points to the limits of framing. In contrast to typical framing manipulations in previous studies, the behavior in question was already very salient to participants and demanded from them daily in this pandemic. By July 2020, they had certainly heard several value-based and/or utility-based appeals to comply with social distancing measures. Therefore, they may have already made up their minds on how the behavior is linked to their personal values and thus may have considered the message as less relevant.

The explorative findings are nevertheless clearly in line with the results from Study 1 and support the theoretical assumptions underlying the hypotheses. The hypothesized processes are based on the theoretical assumption that failing to show a behavior that is seen as linked to the values of an individual threaten their self-regard and identity as a good person. Consistent with this reasoning, participants who saw their moral self-regard as contingent on their social distancing behavior were more inclined to comply and their willingness to comply did not depend as much on the personal cost and perceived efficacy. In addition, Study 2 shows that moral self-regard is similarly related to support of policies to enforce pandemic measures independent of the personal cost and efficacy of the measures.

The findings are limited by their correlative nature and the intercorrelations of self-interest inconsistency and efficacy with moral self-regard. Therefore, it cannot be concluded whether individuals who consider social distancing measures as a matter of values will dismiss information about interests and efficacy or if they appraise information differently. Individuals may have cognitively supported their value perspective on countermeasures at this point in the pandemic and thus resist manipulation in this regard [for similar reasoning, see Maio et al. (2001)]. Experimental evidence is needed to understand whether value consistency moderates the causal effects of utility information.

## Study 3: Value Consistency and the Relevance of a Vaccine's Utility

In a pre-registered experiment, value consistency is examined as a moderator of how factual information causally affects behavioral intentions. At this stage (early December 2020), the focus of attention had shifted from social distancing toward vaccination as the most important measure. Study 3 was conducted shortly after news media reported study results showing 94% effectiveness of the first vaccine in protecting from infection, e.g., BBC News (2020). The willingness of the people to get vaccinated likely depends on whether this is a matter of values for them (H1: The more vaccination is perceived as value-consistent, the higher the willingness to get vaccinated.). Specific information about whether this vaccine is effective to protect themselves (self-interest efficacy) and to protect others (pro-social efficacy) is also likely to affect their willingness. However, based on the previous findings, this information should affect willingness less if vaccination is highly value-consistent (H2/H3). The pre-registration, data, and all original materials are available on the OSF project).

### Method

#### Sample, Recruitment, and Design

A sample of 258 UK residents (162 women, 95 men, one of another gender, was recruited *via* Prolific.ac. Their age ranged from 18 to 71 years (*M* = 36.37, *SD* = 12.67). Two participants were excluded because they failed an attention check. The design is 2 (efficacy of protecting the self: low vs. high) × 2 (efficacy of protecting others: low vs. high) experimental design with a measured moderator (value consistency: ranging from completely inconsistent over neutral to completely consistent). The sample size was approximatively estimated based on the goal to achieve 80% power to detect a small effect of η^2^ = 0.03 in a 2 × 2 × 2 ANCOVA with up to three covariates. This reflects a simplified hypothesis test with a median-split moderator. The actual pre-registered bootstrapped moderation analysis with a continuous moderator (value consistency) is more powerful.

#### Procedure, Manipulation, and Measures

After giving informed consent, participants first reported their values. Within this scale, an attention check item was administered. Then, they completed a measure of the extent to which getting vaccinated against COVID-19 was consistent with their values. Afterward, they received a description of a hypothetical new vaccine that contained the manipulation of both factors, they reported their willingness to get this vaccine, and reasons for this. On the next pages, they answered five manipulation check questions, and demographic questions, which include age, gender, education level, and country of residence. Finally, they answered control questions about how affected participants were by the pandemic.

**Values** were measured with the SSVS (Lindeman and Verkasalo, 2005) to explore the values individuals see as consistent with vaccinations. As an attention check, the following similarly phrased item was included: *ATTENTION (if you read this, select 7 because attention is of supreme importance for this study)*.

**Value consistency** was measured with the three items from Study 1 (α = 0.877). The instructions were adapted to the context of vaccination, such that participants rated the extent to which getting a vaccination against COVID-19 was completely in contrast (−3) to completely in line with their values (+3).

**Experimental manipulations**. Participants were asked to imagine receiving a message that a specific vaccine against COVID-19, but no other options, was now available for them. The vaccine was described as having been tested in several large-scale studies, which had found only very rare and mild side effects. It had been approved by the responsible regulatory agency (Medicines and Healthcare products Regulatory Agency; MHRA) to be administered. Self-interest consistency was operationalized as the alleged effectiveness of the vaccine to protect oneself from infection (60 vs. 90%) and will, thus be referred to as self-interest efficacy for clarity. In contrast to Study 1 and 2, where self-interest consistency was operationalized as ranging from very costly to very beneficial, this operationalization only focuses on varying benefits for the self. Efficacy was operationalized specifically regarding the *prosocial* efficacy that may be particularly relevant for value-driven decision-making. Participants read that the vaccine was 60 vs. 90% effective, respectively, in preventing the spreading of the virus to others. Both factors were operationalized as percentages because this reflects how media outlets reported the first results of studies on new vaccines, e.g., BBC News (2020).

**Willingness to get vaccinated** was measured with three items (How likely would you get vaccinated with this vaccine; How much would you intend to get this vaccine; How willing are you to get this vaccine; α = 0.977) and a Likert scale ranging from 1 (not at all) to 6 (very much).

**Manipulation checks**. Participants rated on a scale ranging from 1 (very little) to 7 (extremely) how effective this vaccine was in protecting them and/or other people around them and how much they would benefit from getting vaccinated and/or others would benefit from them getting vaccinated, for self-interest consistency and prosocial efficacy, respectively. The two items correlated at *r* = 0.788/0.869, *p*s <0.001, respectively. Participants also reported how effective they perceived the described vaccine to be compared to other COVID-19 vaccines and noted in an open-ended field which vaccine producers they knew about.

### Results

#### Preliminary Analyses

An analysis of variance with self-interest efficacy and prosocial efficacy as factors resulted in the expected main effects of self-interest consistency, *F*_(1, 254)_ = 45.98, *p* < 0.001, $\eta_{p}^{2}$ = 0.153, and prosocial efficacy, *F*_(1, 254)_ = 56.53, *p* < 0.001, $\eta_{p}^{2}$ = 0.182, on the respective manipulation checks (*M* and *SD* are reported in the Supplementary Table 3.1). In addition, there was an unexpected main effect of prosocial efficacy on the manipulation check for self-interest consistency, *F*_(1, 254)_ = 3.91, *p* = 0.049, $\eta_{p}^{2}$ = 0.015, indicating that a vaccine that prevented the spreading of the virus to others at higher rate was also perceived as slightly more beneficial for the self than a less pro-socially effective vaccine. There were no significant interaction effect on the manipulation check of self-interest consistency, *F*_(1, 254)_ = 1.049, *p* = 0.307, $\eta_{p}^{2}$ = 0.004, and no unexpected significant effects on the manipulation check of prosocial efficacy, *F*s_(1, 254)_ < 2.3, *p*s > 0.13. A two-way ANOVA of effectiveness compared with other COVID-19 vaccines showed that both self-interest consistency, *F*_(1, 254)_ = 38.77, *p* < 0.001, $\eta_{p}^{2}$ = 0.132, and prosocial efficacy, *F*_(1, 254)_ = 11.62, *p* = 0.001, $\eta_{p}^{2}$ = 0.044, significantly contributed to this perception but did not interact, *F*_(1, 254)_ = 0.24, *p* = 0.625, $\eta_{p}^{2}$ = 0.001.^4^

Gender (dummy-coded), risk group, infection experience, and pandemic effects on the job did not significantly predict willingness to get vaccinated, all βs <0.104, all *p*s > 0.091. Consequently, no covariates were included in the main analyses as pre-registered.

#### Confirmatory Hypothesis Tests and Explorative Analyses

Hypotheses were tested using the PROCESS macro for SPSS (Hayes, 2017). Two moderation models (using 10,000 bootstrapped samples) were calculated with the willingness to get vaccinated as a dependent variable. Model 1 tested whether the effect of self-interest consistency was moderated by value consistency. Model 2 tested whether the effect of prosocial efficacy was moderated by value consistency. The results presented in Table 3 show that Hypothesis 1 can be accepted, as both models show significant main effects of value consistency, such that higher value consistency of vaccination was associated with higher willingness to get vaccinated.

**Table 3:** Moderation of the effect of the efficacy of the vaccine to protect the self (Model 1) or others (Model 2) by value consistency of vaccination.

		**Model 1 (efficacy for self)**			**Model 2 (efficacy for others)**		
		**Coefficient**	**95% CI [LL; UL]**	***p***	**Coefficient**	**95% CI [LL; UL]**	***P***
Efficacy factor		0.679	[0.278; 1.079]	0.001	0.932	[0.530; 1.334]	<0.001
Value consistency		0.578	[0.452; 0.705]	<0.001	0.690	[0.552; 0.827]	<0.001
Interaction		−0.729	[−0.253; 0.108]	0.427	−0.220	[−0.402; −0.039]	0.017
**Conditional effects of experimental factor at different levels of value consistency**							
		**Model 1 (efficacy for self)**			**Model 2 (efficacy for others)**		
**Value consistency**		**Effect**	**95 % CI [LL; UL]**	***p***	**Effect**	**95 % CI [LL; UL]**	***p***
	0	0.679	[0.278; 1.079]	0.001	0.932	[0.530; 1.334]	<0.001
	2	0.533	[0.226; 0.840]	0.001	0.491	[0.188; 0.795]	0.002
	3	0.460	[0.061; 0.858]	0.024	0.271	[-0.124; 0.666]	0.177

*The efficacy factor was coded dichotomously (0, low; 1, high). Value consistency was coded ranging from−3 (vaccination completely inconsistent with values) to 3 (completely consistent). Zero hence reflects a neutral value position*.

The results of Model 1 further show no support for Hypothesis 2, given that individuals were significantly more willing to get the vaccine with 90% than 60% protection of the self, independent of value consistency. Even though the relevance of this information was descriptively slightly lower among those whose values were strongly consistent with vaccination compared to those with a neutral position, this difference was not significant.

**Figure 5:**
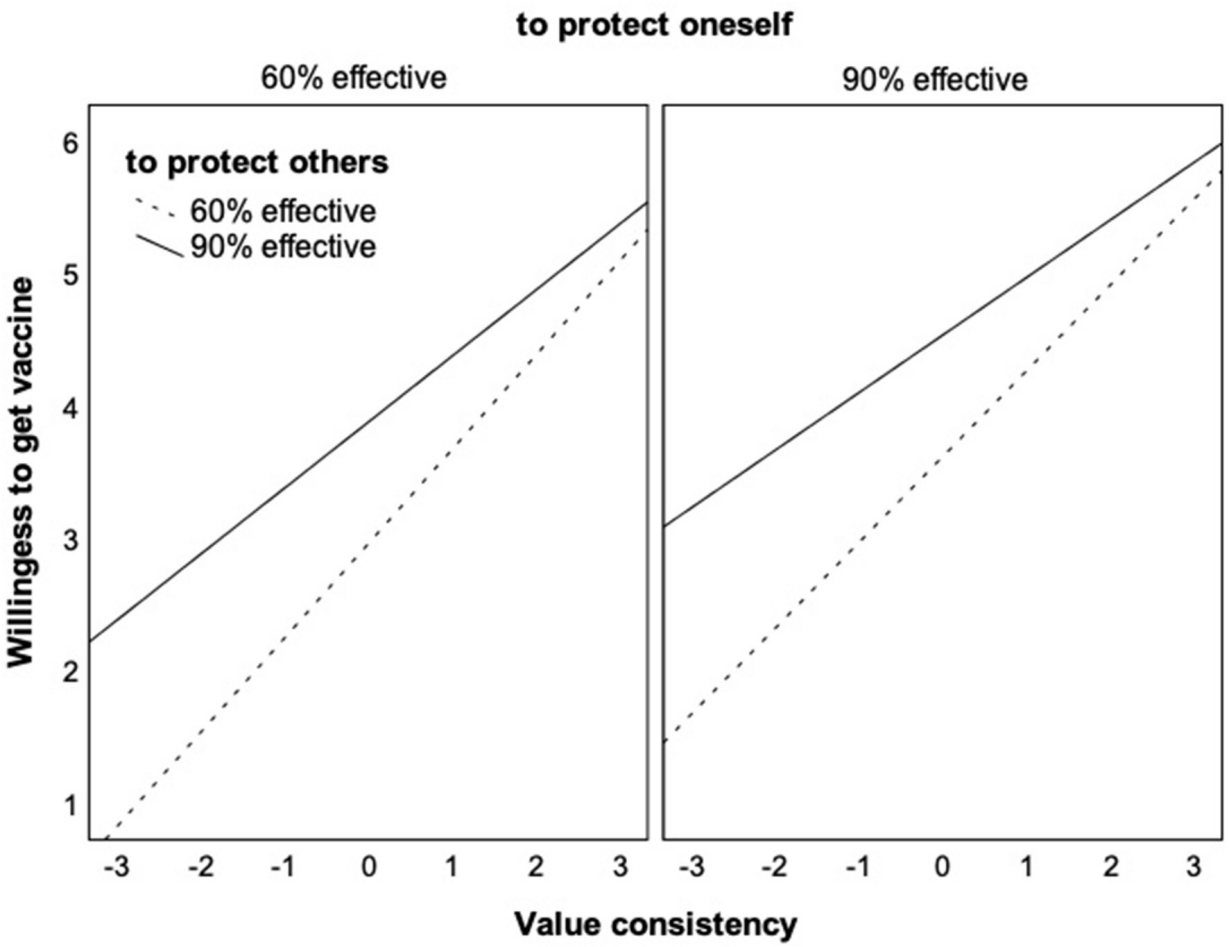
Interaction effects of value consistency with the efficacy to protect the self or others on the willingness to get vaccinated. The figure shows the unique effects of each interaction in the combined model (see Supplementary Table 3.4 for conditional effects).

Hypothesis 3 was supported by the significant interaction in Model 2. Even though individuals overall were more willing to get the vaccine that was 90% than 60% effective in preventing transmission of the virus to other people, this factor was less relevant the more individuals saw vaccination as value-consistent. Among those with high-value consistency, there was no longer a significant difference between the 90 and 60% condition—they were very willing to get vaccinated even with the less effective vaccine. In addition, this study calculated a combined model that tested both interactions and experimental factors at the same time. The results are virtually the same as in the separate models, which are fully reported in the Supplementary Table 3.4. Figure 5 illustrates the combined results.

Explorative analyses of the correlations of the outcomes with specific value types show that different values seem to be associated with vaccination that with the social distancing measures in Study 1 and 2. Only power, *r* = −0.197, *p* = 0.001, and tradition, *r* = −0.188, *p* = 0.002, correlate significantly, and negatively, with this outcome. Value consistency of vaccination correlates significantly with tradition, *r* = −0.266, *p* < 0.001, and conformity, *r* = −0.159, *p* = 0.010.

### Discussion

Study 3 confirmed that the extent to which vaccination is seen as consistent with personal values strongly predicts the willingness of a person to get vaccinated. In addition, the findings confirmed that higher value consistency makes it less relevant whether the vaccine is only moderately or highly effective to protect other people. However, higher value consistency did not make it less relevant whether the vaccine was moderately or highly effective to protect the self (its self-interest efficacy). The operationalization of the factor self-interest efficacy may have been limited by only varying different levels of benefits and did not consider costs like the previous studies.

## General Discussion

### Summary and Limitations

In three studies, it was tested whether individuals are more inclined to follow health measures that they perceive to be linked to and consistent with their personal values. In addition, the studies examined for the first time whether stronger perceptions of value consistency come with an intention to follow the health measure no matter what costs and benefits it brings for the self, and no matter how effectively it can curb the spreading of the virus.

Study 1 provides correlative support of the effect of value consistency on social distancing behaviors and intentions at the beginning of the pandemic in March 2020. The results also confirm the predicted patterns of moderation: perceiving social distancing as more or less costly only explained the distancing intentions of individuals if they saw it as a value-neutral behavior. And perceiving their social distancing behavior as more or less effective in the pandemic also only explained value-neutral of the distancing intentions of the individuals.

Study 2 failed to show that these value consistency effects could be experimentally induced by a public appeal framed as value-based rather than utility-based, most likely because appeals of any framing were very salient anyway back in July 2020, and the perceptions of value-relevance may already have been established among most participants. Nevertheless, exploratory analyses show that the extent to which following social distancing behavior was perceived as important for the moral self-regard of an individual similarly explained behavioral intentions, moderated the personal cost–behavioral intentions, as well as the efficacy–behavioral intentions relationships. In addition, Study 2 showed that these patterns of moral self-regard and utility-based considerations also extended to other outcomes besides behavioral intentions: support of policies to counteract the pandemic, e.g., lockdowns, and the devaluation of others who transgress social distancing guidelines.

In Study 3, the focus was on vaccination intentions and the utility-based information in the form of varying percentages of protection of the self and protection of others was experimentally varied. The findings show that both utility factors significantly predict the willingness to get vaccinated but the value consistency of COVID-19 vaccination of the individuals does as well. Regarding the moderation, the results confirm that individuals who see COVID-19 vaccination as more value consistent wanted to get a vaccine whether it protected others more or less. However, value consistency does not moderate the effect of protection of the self. This seems to contrast the findings of Study 1 and 2, where higher value consistency (Study 1) or moral self-regard (Study 2) moderates the effects of self-interest consistency on behavioral intentions. The absence of a similar pattern may be due to the different operationalizations of self-interest efficacy and self-interest consistency. Importantly, the experimental manipulation in Study 3 varied only the potential benefits of the vaccine, whereas Study 1 and 2 measured variations in the experienced benefits vs. costs of social distancing, which was mostly seen as costly. Important values may particularly serve a function to motivate individuals to make sacrifices to do them justice (Berns et al., 2012; Atran et al., 2014; Pretus et al., 2018). Personal benefits may just represent an additional reward that interacts less with values. The utility-based effects also were generally stronger in Study 3 compared with Study 1 and 2. This may be because the utility-based information was experimentally induced as specific factual estimates, rather than uncertain and multi-faceted subjective estimates. An explanation for this difference between the findings thus may be those values maybe even more important for guiding behaviors under uncertainty. Future research is needed to examine the role of the uncertainty of utility-based information for value-relevant behaviors.

A limitation of this research is that most of the findings are correlative. The factor could not be successfully manipulated experimentally in Study 2. Therefore, it cannot be excluded as a possibility that the measures of value consistency and consequences for self-regard at least partly reflect *post-hoc* justifications of behaviors and intentions that may be costly. However, since value consistency in Study 1 and 3 was measured at the beginning of the study, and the dependent measures explicitly referred to the intention of willingness for future behavior, it seems unlikely that this is mainly responsible for the findings. In addition, the absence of a framing effect in Study 2 further implies how difficult it is to change value-related beliefs once reasoning for them has been established (Maio and Olson, 1998; Schuster et al., 2019).

A second limitation of this work is that the samples include only a few individuals who considered the health measures as inconsistent with their values. It remains an open question whether stronger negative value relevance would also lead to disregard of utility information. Such an effect could, for instance, be involved in the costly protests of some groups and individuals against health measures they see at odds with their autonomy, e.g., shop owners who open despite lockdown. On the other hand, the high prevalence of individuals who see health measures as value consistent in the present work is a positive finding in itself as it relates to higher compliance.

A third limitation of this work may be that it cannot necessarily be generalized beyond European or North American countries. Previous research shows that self-transcendence and conservation values, which are most relevant for the health measures examined here, play an almost non-existent role in guiding consistent behaviors in countries with tight norms (Elster and Gelfand, 2020). Future research could examine if, in these countries, the norms concerning health behaviors have a similar moderating effect of utility-based considerations.

### Implications for Future Research and Theory Development on Value-Guided Behavior

The present findings provide new insights into the role of personal values for planned behavior and decision-making. The presented studies provide the first evidence of an interaction effect of guidance by values and utility while it is well-known that values guide behaviors even if they might be effortful or costly (Karp, 1996; Sagiv et al., 2011) and that great personal sacrifices tend to be justified with sacred values (Berns et al., 2012; Atran et al., 2014). Being guided by values seems to entail an at least partial disregard of utility-based information. Interestingly, this refers to both utility in terms of self-interest as well as utility for other people or overall society. This interpretation is in line with findings from other, methodologically different studies. First, negotiation experiments show that value-driven negotiators disregard payoff information (Stöckli and Tanner, 2014; Schuster et al., 2020). Second, moral dilemma research shows that people facing risky choices tend to disregard higher moral expectancy values (Zlatev et al., 2020). Third, neurological studies found that values operate through a brain system which is separate from the utility system (Berns et al., 2012). Therefore, the present findings are highly relevant for further theorizing of the psychological processes by which values motivate behaviors. Current models, such as the value-identity-personal norms model (Ruepert et al., 2016), need to be further developed by integrating interactions between value-based and utility-based predictors.

In further forwarding the theory building in this area, the present findings provide an important first step for experimental studies of value consistency-utility interactions on behavior in other domains besides health measures in this pandemic. In other contexts, where the relevance of values for a specific behavior is less salient and open to interpretation, e.g., behaviors with little known environmental impact, it could be more effective to manipulate the value consistency of individuals. Another important question to be resolved is whether value consistency leads to disregard only of cost or also of benefits and whether the level of uncertainty of utility-related information also matters.

### Practical Implications for Health Measures the COVID-19 Pandemic

Even though the causality of the effect of value framing in Study 2 was not supported, the high levels of value consistency and relevance for self-regard is most likely a result of what we know about the effect of the pandemic on society and vulnerable groups and of how this has been communicated by authorities. The health measures probably would have seemed less a matter of benevolence and security values if all the information about the Coronavirus had been purely utility-based, e.g., the cost for the health care system, the lost workforce due to infection, etc., and if there had not been pictures showing the exhaustion of nurses and stacks of coffins that illustrated the moral aspects of the crisis.

Given the sacrifices required from the public to curb the pandemic, the findings of these studies indirectly imply that world leaders may have been most reasonable and effective in their appeals to values and morality when demanding lockdown compliance or self-directed distancing. Nevertheless, the present research also points to the ambivalent nature of presenting pandemic behavior as a matter of values. It is certainly not desirable that information about the effectiveness of a measure is disregarded or that costs and benefits of a measure, e.g., mental health problems and suicide risk (McIntyre and Lee, 2020; Usher et al., 2020; Tanaka and Okamoto, 2021), increased flexibility in-home office (Alon et al., 2020, p. 19), are not weighed against their impact on flattening the curve.

In addition, the present work implies that the link between pandemic health measure compliance and values might lead to the moral outrage the Sacred Value Protection Model predicts (Tetlock et al., 2000; Tetlock, 2003). Study 2 showed that individuals who see health measures as strongly relevant for their moral self-view furthermore tend to devalue others who do not comply as unconditionally as they see fit, even for transgressions on less effective and more costly measures. Such negative judgments and scolding could potentially increase social conflict and polarization about health measures. The findings on the support of policies to enforce social distancing measures with less consideration of utility-based information also could point to a risk that the constitutional principle of proportionality may be disregarded if things become a matter of values. These tendencies are particularly problematic for parts of the society that see at least some of the measures as being against their values. Even though in the present samples this was only a small minority, this may matter in practice. If this group is signaled that social distancing and getting a vaccination is a matter of a sense of morality that they do not share, reactance is likely, particularly if they feel–in part correctly–that this moralized view disregards important facts.

Yet even if politicians and scientists would appeal less to values and more to reason, the existential nature of the pandemic and the human lives endangered in it may represent an obvious and important link of measures to curb the spreading of the virus to sacred values. Previous research on sacred values suggests particularly two ways how individuals can be led to consider and weigh the trade-offs in a more rational manner. The first way consists in reframing the perception of how the value in question, and other similarly important values, can be afforded and affirmed [for examples from sacred value conflicts, see Atran and Axelrod (2008)]. For instance, it would be helpful to acknowledge that to act morally in a pandemic, may not be the only way to cut all personal contacts. It would also be safe to keep a few personal contacts if they mutually decided to restrict other contacts. Or one could safely visit vulnerable grandparents for holidays after self-isolating for 2 weeks.

The other way consists in making clear that in a global pandemic, harm is unavoidable (Berman and Kupor, 2020). Trade-offs between different values and value-related goals are unavoidable, thus we may allow ourselves to carefully consider which trade-offs we want to make (Tetlock et al., 2000; Tetlock, 2003) based on all the information about costs for the self and others and the best estimates of the efficacy and potential benefits of each health measure.

## Conclusion

The findings of three studies support that the individuals are clearly more motivated to follow health measures that they perceive as a matter of their core values and moral identity. Nevertheless, it might be the wrong conclusion to foster compliance by discussing health measures as a matter of values rather than rational arguments because it seems to make people disregard costs and varying efficacies of measures and become harsher in their judgment of others. Rather than following health recommendations as a moral principle, individuals should try to particularly implement measures that are very effective (such as wearing masks and getting vaccinated) and also consider potential costs of compliance (e.g., for their mental health). Sometimes a walk in the park with a friend a few steps apart may do more good than harm and be rationally better than social distancing at all costs.

## Data Availability Statement

I thank Jakob Dickhaut, Julia Dehner, Clarissa Leuthold, and Marcel Hartmann for their help with the preparation of the study materials and the supplementary online materials. The original study materials, pre-registrations, and data can be accessed via the osf-project under this link https://osf.io/fxv83/.

## Ethics Statement

Voluntary approval by the Leuphana University Ethics committee was sought and granted for Study 2 (for the others, even less critical, no approval was sought). In Germany, no ethics approval is required for non-clinical psychology studies. Written informed consent from the participants' legal guardian/next of kin was not required to participate in this study in accordance with the national legislation and the institutional requirements.

## Author's Note

Correspondence address: Institute of Psychology, Universitätsallee 1, 21335 Lüneburg, Germany, carolin.schuster@leuphana.de. I thank Jakob Dickhaut, Julia Dehner, and Marcel Hartmann for their help with the preparation of the study materials and the supplementary online materials.

## Author Contributions

The author confirms being the sole contributor of this work and has approved it for publication.

## Conflict of Interest

The author declares that the research was conducted in the absence of any commercial or financial relationships that could be construed as a potential conflict of interest.

## Publisher's Note

All claims expressed in this article are solely those of the authors and do not necessarily represent those of their affiliated organizations, or those of the publisher, the editors and the reviewers. Any product that may be evaluated in this article, or claim that may be made by its manufacturer, is not guaranteed or endorsed by the publisher.

## Footnotes

^1^Not including covariates lead to very similar results and the same conclusions (Supplementary Table 2.8).

^2^Excluding participants who consider the relevant value types the appeal is framed to be driven by–benevolence and universalism– as not very important in their lives, i.e., excluding 21 participants who rated these values <4, did not lead to different conclusions (see Supplementary Table 2.5). Therefore, the lack of an effect cannot be attributed to an appeal that was inconsistent with the values of the sample.

^3^In this analysis, age, gender, and infections were not included as covariates because they were not correlated with these outcomes (Supplementary Material 2.2). However, the results remain robust when including these (see Supplementary Material 2.9).

^4^Diverging from the pre-registered procedure, I did not include education (because of its ordinal scaling) and country of residence (because all participants were from the UK).
