# Supplementary material for: Following Health Measures in the Pandemic: A Matter of Values?
Source: Front Psychol. 2021 Sep 14;12:731799. doi: 10.3389/fpsyg.2021.731799 (PMC8477035; doi:10.3389/fpsyg.2021.731799)
Supplement: Supplementary file 1 [file Data_Sheet_1.pdf]

Supplemental Online Material for the Article "Following Health Measures in the Pandemic: A Matter of Values?"

Contents

Contents 1

1. Supplemental materials for Study 1 2
   1.1. Combining the items for the social distancing behavior scale 2
   Table S1.1. Descriptive statistics of social distancing behaviors 2
   1.2. Descriptive Statistics and Intercorrelations 3
   Table S1.2. Scale means and intercorrelations 3
   1.3. Main moderation models without covariates 3
   Table S1.2. Moderation of interest consistency and efficacy effects on social distancing by value consistency 3
   1.4. Exploratory analyses with exclusion of participants with unreasonably high efficacy 4
   Table S1.3. Moderation model with efficacy cut-off 4
   1.5. Exploratory analyses of perceived danger effects on social distancing 4
   Table S1.4. Moderation of perceived danger effect on social distancing by value consistency. 4
   1.6. Exploratory Analyses of Frequencies of Values 5
   Figure S1.5. Relative frequencies of value types named as associated with value consistency and inconsistency of social distancing demands. 5

2. Supplemental materials for Study 2 6
   2.1. Additional information on the measures 6
   2.2. Correlations of social distancing outcomes with values 6
   Table S2.1. Correlations of Social Distancing Outcomes with Values 6
   2.3. Intercorrelations 7
   Table S2.2. Scale means and intercorrelations (N = 340) 7
   2.4. Pre-registered analyses 8
   Table S2.3. Interaction effects of framing with self-interest consistency and efficacy 8
   Table S2.4. Cross-level effects of framing and measure-specific predictors 8
   2.5. Framing effects after exclusion of individuals with inconsistent value profiles 9
   Table S2.5. Framing effects after exclusion of individuals with inconsistent value profiles 9
   2.6. Means and SDs of outcome variables and self-regard by framing condition 9
   Table S2.6. Means and SDs of outcome variables and self-regard by framing condition 9
   2.7. Exploratory analyses: policy support and devaluation of transgressors 9
   2.8. Robustness of results without/with covariates 10
   Table S2.8. Social distancing intentions as a function of the predictors without the covariates 10
   Table S2.9. Policy support and devaluation as a function of the predictors with covariates 10

3. Supplemental materials for Study 3 11
   3.1. Means and standard deviations by experimental condition 11
   Table S3.1. Means and standard deviations by experimental condition 11
   3.2. Correlations of outcomes with values 11
   Table S3.2. Correlations of outcomes with values 11
   3.3. Model 3 – Combined Model 11
   Table S3.4. Main and interaction effects of value consistency and experimental factors 11

1. Supplemental materials for Study 1

1.1. Combining the items for the social distancing behavior scale

Table S1.1 of the descriptive statistics of the specific social distancing items showed that a considerable percentage of participants indicated that the demand to avoid contact with colleagues or clients did not apply to them. In addition, the items have different difficulties, such that combining them ignoring missing values could reduce reliability. To combine the items to a scale, these two items were thus ignored and only participants who completed all other items were included in the analyses of this measure. Separate scales for the past and coming week correlated highly, $r = .737$, thus the five items of each were summed to one social distancing behavior scale ($\alpha = .87$) ranging from 0 (none of them followed at all in any week) to 30 (all behaviors completely followed in both weeks).

Table S1.1. Descriptive statistics of social distancing behaviors

Behavior	NA %	M_{last}	SD_{last}	M_{next}	SD_{next}
hold at least 2 m distance to others	2.12	2.14	0.58	2.46	0.62
avoid touching, e.g. shaking hands	2.12	2.74	0.56	2.82	0.49
avoid being in the same room with colleagues at work	36.40	2.25	1.00	2.43	0.92
avoid contact with clients or customers at work	44.46	2.43	0.90	2.62	0.80
avoid private events with more than 5 persons	3.18	2.82	0.52	2.80	0.59
avoid private meetings, e.g. with friends	3.18	2.30	0.81	2.37	0.85
avoid visiting public places like restaurants and gyms	3.89	2.88	0.42	2.90	0.38

Note. The items are coded such that 0 means measure was “not followed at all” and 3 it was “followed completely”.

1.2. Descriptive Statistics and Intercorrelations

Table S1.2. Scale means and intercorrelations

		1	2	3	4	5	6	7	8
	M	26.38	17.18	1.42	0.06	11.71	15.87	14.10	33.01
	SD	3.85	2.78	1.52	1.56	5.81	4.71	4.81	13.82
	N	261	278	280	280	266	277	276	276
1. Distancing behaviors	r								
2. Distancing overall		.666**							
3. Value consistency		.299**	.322**						
4. Interest consistency		.189**	.211**	.390**					
5. Behavioral control		.038	.002	.233**	.197**				
6. Efficacy		.250**	.393**	.320**	.285**	.085			
7. Danger of virus		.301**	.301**	.356**	.204**	.188**	.330**		
8. Age		.207**	.217**	.055	.110	.083	.129*	.214**	
9. Risk group (dummy)		.148*	.153*	-.018	.081	.041	.141*	.158**	.351**
10. Gender (dummy)		-.084	.013	-.017	.085	.094	-.007	-.096	.162**
11. Day		-.276**	-.224**	-.125*	-,120*	-.067	-,164**	-.261**	-.539**

Note. * $p < .05$. ** $p < .01$. The variable day is a variable from the meta-data of the survey indicating the day since data collection started. Higher values thus also indicate that the time of social distancing has already been longer. The complete output with exact *p*s and pairwise *N*s is available in the osf-project.

Table S1.2 shows interest and value consistency, as well as perceived danger and efficacy, correlate positively with social distancing. In addition, being a member of a high-risk group for Covid19, age, and the day of data collection is related to social distancing.

1.3. Main moderation models without covariates

The main hypothesis test in the manuscript are reported with the covariates age, risk group, and day of survey (i.e., later also means longer time of social distancing period). Table S1.2 shows the same analyses without these covariates.

Table S1.2. Moderation of interest consistency and efficacy effects on social distancing by value consistency

	Social distancing behaviors			Social distancing summary		
	coefficient	95% CI [LL; UL]	p	coefficient	95% CI [LL; UL]	p
H1 model						
Interest consistency	0.59	[0.148; 1.024]	.01	0.25	[-0.041; 0.547]	.09
Value consistency	0.54	[0.201; 0.884]	.00	0.48	[0.248; 0.708]	.00
Interaction	-0.21	[-0.397; -0.031]	.02	-0.06	[-0.287; 0.061]	.32
H2 model						
Efficacy	0.23	[0.114; 0.343]	.00	0.25	[0.185; 0.325]	.00
Value consistency	2.10	[1.128; 3.076]	.00	1.75	[1.135; 2.356]	.00
Interaction	-0.09	[-0.154; -0.034]	.00	-0.09	[-0.124; -0.049]	.00

Note. The Table displays the results of bootstrapped models of the effects of (1) interest consistency and (2) efficacy on social distancing compliance, and their moderation by value consistency. Efficacy is coded ranging from 0 (no efficacy) to 20 (complete efficacy). Interest and value consistency of social distancing are coded such that 0 indicates neutral positions, -3 the highest inconsistency and +3 the highest consistency.

1.4. Exploratory analyses with exclusion of participants with unreasonably high efficacy

Since many participants reported the highest possible value of efficacy, I tested if the variables main effect and the moderation by value consistency held if those participants were included. Therefore, a variable was created (selfeff_below100) in which participants who reported 100% efficacy of their own behavior to curb the outbreak were coded as missing values. Table S1.3 shows that the results were the same.

Table S1.3. Moderation model with efficacy cut-off

	Social distancing behaviors			Social distancing summary		
	coefficient	95% CI [LL; UL]	p	coefficient	95% CI [LL; UL]	p
Efficacy<100	0.12	[-0.035; 0.268]	.13	0.22	[0.132; 0.314]	.00
Value consistency	2.11	[0.917; 3.311]	.00	1.91	[1.182; 2.632]	.00
Interaction	-0.08	[-0.166; -0.003]	.04	-0.10	[-0.148; -0.048]	.00

Note. The table displays the results of bootstrapped models of the effects of perceived danger of corona virus on social distancing compliance, and their moderation by value consistency. Efficacy is coded ranging from 0 (no efficacy) to 20 (complete efficacy), but all participants that had reported 100% (i.e., 20) were coded as missing values.

1.5. Exploratory analyses of perceived danger effects on social distancing

As perceived danger also correlated positively with reported social distancing behaviors, I tested whether this relationship was also moderated by value consistency. The results are reported in Table S1.4.

Table S1.4. Moderation of perceived danger effect on social distancing by value consistency

	Social distancing behaviors			Social distancing summary		
	coefficient	95% CI [LL; UL]	p	coefficient	95% CI [LL; UL]	p
Danger	0.20	[0.094; 0.310]	.00	0.11	[0.035; 0.185]	.00
Value consistency	1.62	[0.812; 2.418]	.00	0.93	[0.385; 1.483]	.00
Interaction	-0.08	[-0.131; -0.022]	.01	-0.04	[-0.074; 0.002]	.06
Risk group	1.14	[-0.123; 2.400]	.08	0.52	[-0.317; 1.356]	.22
Day	-0.06	[-0.096; -0.020]	.00	-0.02	[-0.046; 0.007]	.14
Age	0.00	[-0.039; 0.044]	.90	-0.03	[-0.003; 0.054]	.08

Note. The table displays the results of bootstrapped models of the effects of perceived danger of corona virus on social distancing compliance, and their moderation by value consistency. Perceived danger is ranging from 0 (no danger at all) to 20 (highest possible danger)

1.6. Exploratory Analyses of Frequencies of Values

The frequencies of values marked as relevant by participants who saw social distancing as consistent or inconsistent with their values provides a better understanding of the kind of value concerns that participants had. Figure 1 shows that those reporting value inconsistency most frequently named self-direction as the value type involved, while those reporting value consistency most frequently named security, universalism, and benevolence.

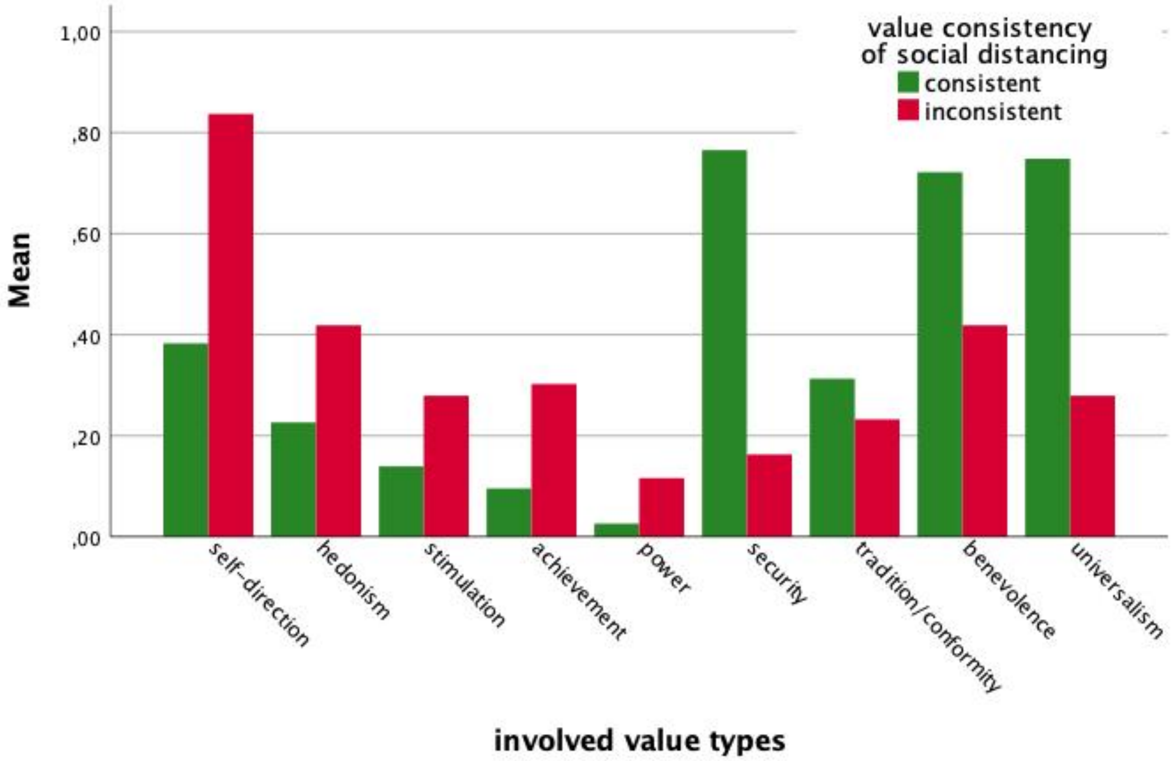

Figure S1.5. Relative frequencies of value types named as associated with value consistency and inconsistency of social distancing demands.

The y-axis displays means of dummy variables where 0 = not quoted and 1 is quoted. Therefore, the means can be read as proportions of participants in the group that named this value.

2. Supplemental materials for Study 2

2.1. Additional information on the measures

The measure of regulatory focus was introduced with “When you think about the reasons to follow social distancing measures, do you focus more on avoiding negative outcomes or on obtaining positive outcomes…” following the three items “…with regard to the further development of the pandemic?”, “…with regard to your self-view as a decent person?”, “…with regard to your personal situation and the consequences of your behavior?”. They answered on a 6-point scale from 1 (prevention focus) to 6 (promotion focus).
The items of the norm measure, which was not correctly recorded, were: “Most people who are important to me think that I..” should comply (1) to shouldn’t comply (6, reverse coded), “Most people whose opinions I value would approve of me complying with the pandemic countermeasures.” rated from strongly disagree (1) to strongly agree (6), and “If I don’t follow the measures, other people will react…” positively (1) to negatively (6). For each item, there was an additional option to indicate that other people were indifferent to the participants’ compliance, which was coded as missing value.

2.2. Correlations of social distancing outcomes with values

Table S2.1. Correlations of Social Distancing Outcomes with Values

	Distancing summary	Mean distancing intentions	Policy support	Devaluation of transgressors	Self-regard
1. Power	-.085	-.177**	-.168**	.044	-.115*
2. Achievement	.007	-.014	.049	.124*	.128*
3. Hedonism	-.110*	-.115*	-.035	-.010	-.024
4. Stimulation	-.008	.019	-.007	.072	-.010
5. Self-Direction	.012	-.008	.034	.023	.070
6. Universalism	.048	.089	.178**	.160**	.193**
7. Benevolence	.203**	.246**	.193**	.163**	.240**
8. Tradition	.177**	.180**	.011	.028	.047
9. Conformity	.103	.076	-.085	.038	.065
10. Security	.172**	.103	.083	.117*	.121*

Note. * p < .05. **p<.01.

2.3. Intercorrelations

Table S2.2. Scale means and intercorrelations (N = 340)

		1	2	3	4	5	6	7	8	9	10	11	12	13
	M	81.37	5.53	4.36	4.16	0.99	4.99	4.49	5.69	4.01	.08	--	--	26.59
	SD	15.85	.93	1.28	1.06	1.13	1.11	1.37	.93	1.26	.271			8.41
1. Distancing summary	r													
2. M. distancing intentions		.650**												
3. Policy support		.501**	.567**											
4. Devaluation		.431**	.437**	.529**										
5. Self-interest consistency		.276**	.387**	.405**	.243**									
6. Efficacy		.354**	.497**	.448**	.386**	.394**								
7. Self-Regard		.365**	.411**	.474**	.568**	.244**	.344**							
8. Behavioral Control		.217**	.365**	.262**	.172**	.326**	.326**	.199**						
9. Regulatory Focus		.068	.110*	.033	.120*	.022	.148**	.029	.262**					
10. Infection		.092	.117*	.129*	.142**	.041	.080	.109*	.074	.028				
11. Risk group (dummy)		.032	.076	-.027	-.080	.049	.054	.012	.092	.085	.006			
12. Gender (dummy)		.093	.150**	.067	.118*	-.016	.131*	.227**	.154**	-.041	.027	.172**		
13. Age		.134*	.165**	.025	.035	-.025	.062	-.003	.195**	.049	-.002	.176**	.106	
14. Job affected		.058	.037	-.089	.075	-.038	.050	.011	.074	.071	.052	.031	.011	.055

Note. * $p < .05$. ** $p < .01$.

2.4. Pre-registered analyses

Table S2.3. Interaction effects of framing with self-interest consistency and efficacy

	Mean distancing intentions			Distancing summary		
	coefficient	95% CI [LL; UL]	*p*	coefficient	95% CI [LL; UL]	*p*
H1 model						
Self-interest consistency	0.276	[0.174; 0.379]	<.001	3.274	[1.408; 5.141]	.001
Framing condition	-0.169	[-0.409; 0.071]	.167	-3.261	[-7.620; 1.097]	.142
Interaction	0.109	[-0.052; 0.270]	.184	1.595	[-1.333; 4.523]	.285
Age	0.018	[0.007; 0.028]	.001	0.254	[0.060; 0.447]	.010
Gender	0.275	[0.086; 0.464]	.004	2.821	[-0.607; 6-248]	.106
Infection	0.187	[-0.021; 0395]	.078	2.632	[-1.141; 6.406]	.171
H2 model						
Efficacy	0.378	[0.276; 0.481]	<.001	4.251	[2.340; 6.162]	<.001
Framing condition	-0.319	[-1.046; 0.409]	.389	-8.755	[-22.272; 4.763]	.204
Interaction	0.047	[-0.108; 0.203]	.550	1.444	[-1.451; 4.339]	.327
Age	0.014	[0.004; 0.024]	.008	0.206	[0.016; 0.395]	.034
Gender	0.135	[-0.046; 0.317]	.144	1.074	[-2.298; 4.447]	.531
Infection	0.149	[-0.050; 0349]	.142	2.173	[-1534; 5.880]	.250

Table S2.4. Cross-level effects of framing and measure-specific predictors

Intended compliance with measure		
	coefficient	*p*
**H1 model**		
Level 1 (measure)		
Self-interest consistency of measure	0.33	.000
Level 2 (person)		
Framing condition	-0.03	.738
Age	0.02	.060
Gender	0.25	.015
Infection	0.23	.045
Cross-level interaction		
Self-interest consistency *Framing condition	0.04	.366
**H2 model**		
Level 1 (measure)		
Efficacy of measure	0.41	.000
Level 2 (person)		
Framing condition	-0.03	.733
Age	0.02	.004
Gender	0.26	.013
Infection	0.22	.059
Cross-level interaction		
Efficacy *Framing condition	0.12	.057

Note: Efficacy and Self-interest consistency are centered to the grand mean.

2.5. Framing effects after exclusion of individuals with inconsistent value profiles

The following analysis excludes participants who consider the relevant value types the appeal is framed to be driven by–benevolence and universalism– as not very important in their lives (i.e., excluding 21 participants who rated these values < 4). As the table shows, the results are very similar to those including these participants. Therefore, the lack of an effect cannot be attributed to an appeal that was inconsistent with the sample's values.

Table S2.5. Framing effects after exclusion of individuals with inconsistent value profiles

	Mean distancing intentions			Distancing summary measure		
Combined Model	B	95% CI [LL; UL]	p	B	95% CI [LL; UL]	p
Efficacy (a)	0.314	[0.208; 0.419]	<.001	3.453	[1.446; 5.459]	.001
Self-interest consistency (b)	0.177	[0.075; 0.278]	.001	2.181	[0.259; 4.103]	.026
Framing (c)	-0.173	[-0.894; 0.548]	.638	-7.084	[-20.769; 6.601]	.309
Interaction a*c	0.002	[0.164; 0.168]	.982	0.937	[-2.216; 4.091]	.559
Interaction b*c	0.058	[-0.107; 0.224]	.487	0.599	[-2.535; 3.734]	.707
Gender	0.173	[-0.004; 0.351]	.059	1.522	[-1.845; 4.889]	.374
Age	0.015	[0.005; 0.025]	.003	0.220	[0.033; 0.408]	.022
Infection	0.138	[0.055, 0.331]	.162	2.036	[-1.629; 5-702]	.275

2.6. Means and SDs of outcome variables and self-regard by framing condition

Table S2.6. Means and SDs of outcome variables and self-regard by framing condition

	Neutral Frame (n = 175)		Value Frame (n = 165)	
	M	SD	M	SD
Mean distancing intentions	5.57	0.94	5.49	0.91
Distancing summary measure	80.09	16.49	78.69	18.23
Self-regard	3.39	1.43	3.59	1.30
Policy support	4.92	0.92	4.87	0.90
Judgment of transgressors	4.14	1.06	4.18	1.06

2.7. Exploratory analyses: policy support and devaluation of transgressors

Table S2.7. Policy support and devaluation as a function of the predictors

	Policy support			Devaluation of transgressors		
	coefficient	95% CI [LL; UL]	p	coefficient	95% CI [LL; UL]	p
Self-interest model						
Self-interest consistency	0.580	[0.377; 0.784]	<.001	0.414	[0.180; 0.649]	.001
Self-regard	0.344	[0.269; 0.420]	<.001	0.491	[0.405; 0.578]	<.001
Interaction	-.094	[-0.148; -0.040]	.001	-0.088	[-0.150; -0.026]	.006
Efficacy model						
Efficacy	0.585	[0.412; 0.757]	<.001	0.435	[0.238; 0.731]	<.001
Self-regard	0.678	[0.457; 0.899]	<.001	0.693	[0.442; 0.944]	<.001
Interaction	-.099	[-0.147; -0.051]	<.001	-0.071	[-0,126; -0.016]	.011

Note: N is larger than in the original model because without gender as a covariates, individuals with missing or non-binary gender are included.

2.8. Robustness of results without/with covariates

Table S2.8. Social distancing intentions as a function of the predictors without the covariates

	Mean distancing intentions			Distancing summary measure		
Self-interest model	coefficient	95% CI [LL; UL]	p	coefficient	95% CI [LL; UL]	p
Self-interest consistency	0.508	[0.290; 0.726]	<.001	7.713	[3.774; 11.652]	<.001
Self-regard	0.289	[0.209; 0.370]	<.001	4.848	[3.393; 6.303]	<.001
Interaction	-0.073	[-0.131; -0.015]	.013	-1.395	[-2.438; -0.353]	.009
Efficacy model	coefficient	95% CI [LL; UL]	p	coefficient	95% CI [LL; UL]	p
Efficacy	0.616	[0.438; 0.793]	<.001	8.321	[5.014; 11.626]	<.001
Self-regard	0.565	[0.338; 0.792]	<.010	9.494	[5.267; 13.722]	<.001
Interaction	-0.086	[-0.136; -0.037]	.001	-1.431	[-2.352; -0.509]	.002

Table S2.9. Policy support and devaluation as a function of the predictors with covariates

	Policy support			Devaluation of transgressors		
	coefficient	95% CI [LL; UL]	*p*	coefficient	95% CI [LL; UL]	*p*
Self-interest model						
Self-interest consistency	0.588	[0.379; 0.797]	<.001	0.437	[0.197; 0.677]	<.001
Self-regard	0.344	[0.267; 0.421]	<.001	0.484	[0.396: 0.572]	<.001
Interaction	-0.96	[-0.152; -0.041]	.001	-0.094	[-0.158; -0.031	.004
Age	0.006	[-0.004; 0.015]	.254	0.007	[-0.004; 0.019]	.185
Gender	-0.006	[-0.181; 0.169]	.948	0.023	[-0.178; 0.224]	.823
Infection	0.148	[-0.038; 0.335]	.119	0.174	[-0.040; 0.388]	.111
Efficacy model						
Efficacy	0.578	[0.403; 0.0752]	<.001	0.441	[0.244; 0.640]	<.001
Self-regard	0.676	[0.452; 0.900]	<.001	0.708	[0.454; 0.962	<.001
Interaction	-.098	[-0.147; -0.049]	<.001	-0.076	[-0.131; -0.020]	.008
Age	0.003	[-0.007; 0.012]	.545	0.005	[-0.006; 0.016]	.343
Gender	-0.077	[-0.250; 0.096]	.381	-0.025	[-0.221; 0.172]	.803
Infection	0.144	[-0.041; 0.329]	.127	0.168	[-0.042; 0.378]	.117

3. Supplemental materials for Study 3

3.1. Means and standard deviations by experimental condition

Table S3.1. Means and standard deviations by experimental condition

	60self/60other		90self/60other		60self/90other		90self/90other	
	n = 64		n = 65		n = 64		n = 65	
	M	SD	M	SD	M	SD	M	SD
MC interest consistency	4.13	1.43	5.39	1.13	4.62	1.32	5.55	1.29
MC prosocial efficacy	4.51	1.46	4.91	1.17	5.84	1.28	5.92	1.06
Relative effectiveness	3.52	1.49	4.75	1.46	4.23	1.61	5.29	1.35
Intentions	3.99	1.67	4.83	1.47	4.66	1.53	5.04	1.19

3.2. Correlations of outcomes with values

Table S3.2. Correlations of outcomes with values

	Value Consistency	Intentions
1. Power	-.113	-.197**
2. Achievement	-.069	-.116
3. Hedonism	-.005	-.090
4. Stimulation	.056	.064
5. Self-Direction	.057	.036
6. Universalism	.051	.008
7. Benevolence	.082	.120
8. Tradition	-.266**	-.188**
9. Conformity	-.159*	-.119
10. Security	.008	-.015

Note: * p < .05. ** p <.01.

3.3. Model 3 – Combined Model

Table S3.4. Main and interaction effects of value consistency and experimental factors

	Intention		
	coefficient	95% CI [LL; UL]	*p*
**Combined Model**			
a. Efficacy for self	0.659	[2.734; 1.044]	.001
b. Efficacy for others	0.918	[0.528; 1.311]	<.001
c. Value consistency	0.713	[0.555; 0.871]	<.001
Interaction a*c	-0.064	[-0.238; 0.110]	.470
Interaction b*c	-0.214	[-0.390; -0.037]	.018

Conditional effects of value consistency

		Efficacy to protect the self					
			**90 %**			**60 %**	
		Effect	CI	*p*	Effect	CI	*p*
Efficacy to protect others	**90 %**	0.44	[0.292; 0.579]	<.001	0.50	[0.356; -0.643]	<.001
	**60 %**	0.65	[0.488; 0.811]	<.001	0.71	[0.555; 0.871]	<.001
